# SOX4 promotes beige adipocyte-mediated adaptive thermogenesis by facilitating PRDM16-PPARγ complex

**DOI:** 10.7150/thno.77102

**Published:** 2022-11-07

**Authors:** Huanming Shen, Ting He, Shuai Wang, Lingfeng Hou, Yixin Wei, Yunjia Liu, Chunli Mo, Zehang Zhao, WeiXin You, Huiling Guo, Boan Li

**Affiliations:** 1 State key laboratory of cellular stress biology, innovation center for cell signaling network and engineering research center of molecular diagnostics of the ministry of education, school of life sciences, Xiamen university, Xiamen 361100, Fujian, China.; 2 Lead Contact.

**Keywords:** beige fat, thermogenesis, obesity, PPARγ, SOX4.

## Abstract

Brown and beige fat protect against cold environments and obesity by catabolizing stored energy to generate heat. This process is achieved by controlling thermogenesis-related gene expression and the development of brown/beige fat through the induction of transcription factors, most notably PPARγ. However, the cofactors that induce the expression of thermogenic genes with PPARγ are still not well understood. In this study, we explored the role of SOX4 in adaptive thermogenesis and its relationship with PPARγ.

**Methods:** Whole transcriptome deep sequencing (RNA-seq) analysis of inguinal subcutaneous white adipose tissue (iWAT) after cold stimulation was performed to identify genes with differential expression in mice. Indirect calorimetry detected oxygen consumption rate and heat generation. mRNA levels were analyzed by qPCR assays. Proteins were detected by immunoblotting and immunofluorescence. Interaction of proteins was detected by endogenous and exogenous Co-IP. ChIP-qPCR, FAIRE assay and luciferase reporter assays were used to investigate transcriptional regulation.

**Results:** SOX4 was identified as the main transcriptional effector of thermogenesis. Mice with either adipocyte-specific or UCP1^+^ cells deletion of SOX4 exhibited significant cold intolerance, decreased energy expenditure, and beige adipocyte formation, which was attributed to decreased thermogenic gene expression. In addition, these mice developed obesity on a high-fat diet, with severe hepatic steatosis, insulin resistance, and inflammation. At the cell level, loss of SOX4 from preadipocytes inhibited the development of beige adipocytes, and loss of SOX4 from mature beige adipocytes reduced the expression of thermogenesis-related genes and energy metabolism. Mechanistically, SOX4 stimulated the transcriptional activity of *Ucp1* by binding to PPARγ and activating its transcriptional function. These actions of SOX4 were, at least partly, mediated by recruiting PRDM16 to PPARγ, thus forming a transcriptional complex to elevate the expression of thermogenic genes.

**Conclusion:** SOX4, as a coactivator of PPARγ, drives the thermogenic gene expression program and thermogenesis of beige fat, promoting energy expenditure. It has important physiological significance in resisting cold and obesity.

## Introduction

Adaptive thermogenesis is a mechanism of metabolic heat production in response to external stimuli, which has an important role in regulating energy homeostasis 1. It is essential for maintaining the body core temperature and promoting survival during cold environments 2. Except for muscle shivering thermogenesis, brown and beige adipose tissues are the main part of non-shivering thermogenesis 3-5. The thermogenic activity of brown and beige adipocytes is largely dependent on uncoupling protein 1 (UCP1), a protein located on the inner mitochondrial membrane 6, 7. It generates heat by dissipating the proton gradient generated by the electron transport chain, which uncouples oxidative respiration from ATP synthesis 8, 9. High expression of thermogenic gene *Ucp1* is required for maintaining the high thermogenic activity of brown or beige adipocytes 10. It has been found that UCP1-deficient mice consume less oxygen and are more sensitive to cold, indicating that their thermoregulation is defective 11.

Cold stimulation mainly activates sympathetic nerves to secrete adrenaline, which acts on β3-adrenoceptors of brown and beige adipocytes, triggers cascade regulation of cell signals, and promotes peroxisome proliferator-activated receptors γ (PPARγ) and its coregulatory factors on the transcriptional regulation of thermogenic genes such as *Ucp1* 3. In addition to maintaining the constant body temperature of mammalians, adaptive thermogenesis can be used to consume obese high calories 12-15. Moreover, decreased thermogenesis dependent on adrenergic activation contributes to obesity-proneness 2, 16. Thus, manipulation of thermogenesis could be an effective method to manage obesity.

Beige adipocytes are 'brown-like' cells that develop in white fat of mammalian postnatally in response to various external stimuli, such as chronic cold exposure. They have multilocular and small lipid droplet morphology and dense mitochondria, high expression of UCP1, and high thermogenetic capacity 17, 18. Similar to white adipose cells, beige adipose cells are derived from platelet derivative growth factor receptor-α (PDGFRα^+^) and stem cell antigen 1 (SCA1^+^) positive or myoglobulin heavy chain 11 positives (MyH11^+^) precursor cells 19-22. In addition to de novo differentiation of precursor cells, studies have shown that beige adipose cells can also transdifferentiate directly from mature white fat cells 23, 24. The activation of beige adipose tissue can significantly promote systemic energy consumption 12. Obesity and related metabolic disorders have been observed in mice lacking beige adipocytes 16, 24. Given that beige adipocytes can enhance energy consumption, they have become a new target for treating obesity and improving metabolic phenotypes. However, the regulatory mechanisms involved in the development and functional maintenance of beige fat remain to be further elucidated.

Peroxisome proliferator-activated receptors (PPARγ) are a key switch that determines the fate of cells and differentiation into brown/beige or white adipose cells. PPARγ drives the brown/beige or white adipogenesis transcriptional program together with distinct co-activators and regulators. PPARγ drives the beige adipogenesis transcriptional program together with CCAAT/enhancer-binding protein-β (C/EBPβ), PPARγ coactivator-1α (PGC-1α), and PRDM16 25. The PPARγ/RXRα heterodimer regulates transcription by directly binding to the peroxisome proliferator-activated receptor (PPAR)-responsive element (PPRE) site on the promoter/enhancer region of its target gene 26. Treatment with PPARγ agonists such as thiazolidinediones (TZDs) can induce beige adipogenesis by increasing PRDM16 protein stability, inducing PPARγ deacetylation, and enhancing PRDM16-PPARγ complex formation 27, 28. However, PPARγ agonists may also lead to serious side effects. Thus, exploring new drugs targeting combined therapy to reduce side effects is of importance.

SOX4 is an important development transcription factor that regulates stem cell characteristics, differentiation, and various development pathways. For example, SOX4 is found to be critical for embryonic heart 29, osteoblast development 30, and initiation of neuronal differentiation programs 31. We recently found that SOX4 can inhibit the orientation of mesenchymal stem cells into preadipocytes and reduce the white adipocytes' differentiation potential of preadipocytes (under review). In order to address the physiological role of SOX4 in WAT development, preadipocyte-specific Sox4 transgenic (Pref1-Sox4) mice were generated and fed on high fat diet. We found that Pref1-Sox4 mice exhibit resistant to obesity with high fat diet (HFD). In the metabolic phenotype analysis, the higher thermogenesis in Pref1-Sox4 mice attracted our attention.

In this study, we explored the role of SOX4 in adaptive thermogenesis. We created mouse models in which Adiponectin-Cre and Ucp1-Cre drove SOX4 deletion. Under prolonged cold stimulation, SOX4 deletion significantly inhibited the formation of beige adipocytes, reduced body temperature and efficiency of energy metabolism. SOX4 adipocyte-specific knockout mice were easy to develop obesity and insulin resistance under HFD. Also, knockdown of Sox4 inhibited the differentiation of beige adipocytes and reduced the thermogenic capacity and energy metabolism of mature beige adipocytes *in vitro.* Mechanistically, SOX4 formed a complex with PPARγ and PRDM16, enhanced the binding of PRDM16 to PPARγ, and promoted the transcription of the thermogenic gene *Ucp1*. These data are of great significance for the development of obesity treatment drugs in the future.

## Results

### Cold induces SOX4 expression in thermogenic adipocytes

To address the physiological role of SOX4 in WAT development *in vivo*, we generated Pref1-Sox4 mice and fed them with HFD (under review). We further found that overexpression of Sox4 in preadipocytes reduced the body weight of mice with HFD **(Figure S1A)**, relieved glucose intolerance as well as insulin resistance **(Figure S1B-C)**, and improved the heat production without obvious effect on food intake and locomotor activity **(Figure S1D, E)**, which suggested that SOX4 may regulate thermogenesis in response to certain external stimuli.

**Figure 1:**
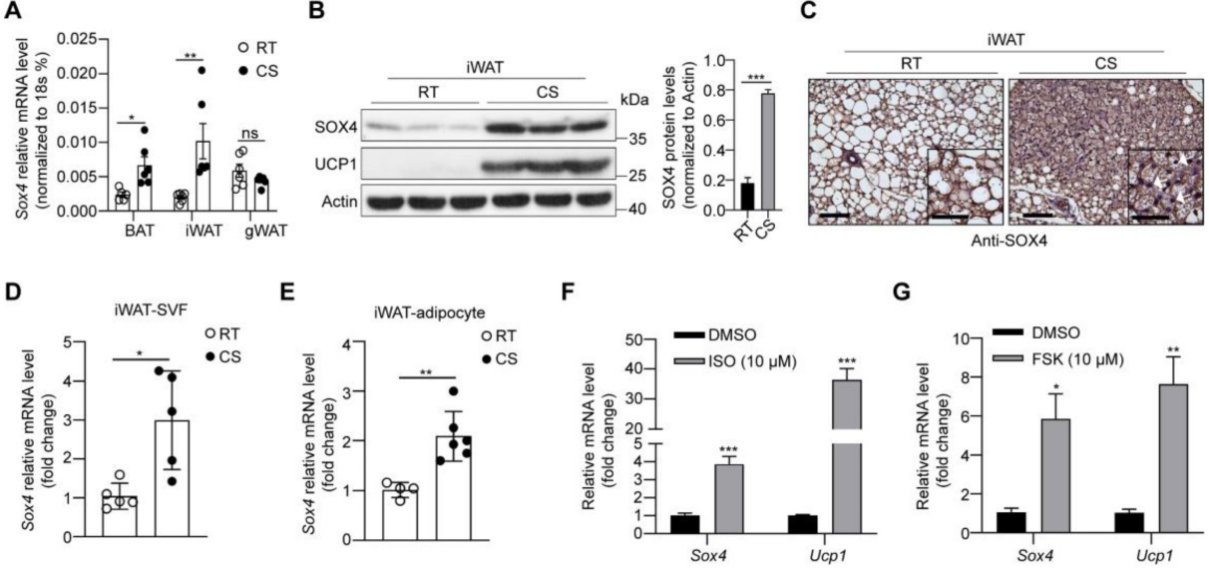
** Cold stimulation induces SOX4 expression.** 10-week male mice were housed at room temperature (RT) or exposed to cold exposure (cold stimulation, CS, 10 °C for one day and then 4 °C for 1 week). BAT, iWAT and gWAT were isolated and subjected to qPCR analysis **(A)** and Western blotting **(B).** The protein levels of SOX4 were quantified with image J (B, right). **(C)** Representative images of SOX4 immunohistochemistry (IHC) of iWATs. Scale bar, 100 µm. Insets show higher magnification, scale bar, 50 µm. **(D)** qPCR analysis of Sox4 mRNA expression in the SVF cells isolated from iWAT. **(E)** qPCR analysis of Sox4 mRNA expression in the adipocytes isolated from iWAT. **(F)** SVF cells isolated from iWAT were differentiated into beige adipocytes* in vitro* as described in method. On day 6, the differentiated beige adipocytes were treated with ISO (isoproterenol) for 4 hr, and then analyzed by qPCR. **(G)** Beige adipocytes differentiated from iWAT SVF cells were treated with FSK (forskolin). 4 hr later, cells were harvested for qPCR analysis.

We first examined the expression of Sox4 in brown adipose tissue (BAT), inguinal subcutaneous white adipose tissue (iWAT) and gonadal white adipose tissue (gWAT). The results showed that protein levels of SOX4 were highest in gWAT and lowest in iWAT (**Figure S2A**). Cold exposure is an important factor inducing adaptive thermogenesis of adipose tissue. We therefore examined whether the expression of SOX4 in adipose tissue responds to prolonged cold exposure. WT mice were subjected to 10 °C for 3 days for cold adaptation and then 4 °C for 7 days. The expressions of SOX4 in BAT, iWAT and gWAT were analyzed. The results showed that the abundance of SOX4 increased most in iWAT, and moderately in BAT, and had no significant change in gWAT with prolonged cold exposure** (Figure 1A, 1B, Figure S2B, S2C)**. Immunohistochemical experiments further confirmed that prolonged cold exposure stimulated iWAT to develop beige adipocytes and enhanced the expression of SOX4 **(Figure 1C)**.

Furthermore, we isolated mature adipocytes and stromal-vascular fraction (SVF) cells from iWAT of WT mice and found that *Sox4* mRNA levels were higher at 4 °C than at 25 °C in both fractions **(Figure 1D-E)**. We also isolated SVF cells and differentiated them into mature beige adipocytes* in vitro*. Treatment of these cells with isoproterenol or forskolin which could mimic cold stimulation caused a significant elevation in *Sox4* mRNA levels **(Figure 1F-G)**. These results showed that cold stimulation significantly induced the expression of SOX4 in thermogenic adipose tissue.

### Adipose tissue-specific or thermogenic adipose tissue-specific SOX4 KO reduced cold tolerance, energy metabolism, and thermogenic function of beige adipocytes

**Figure 2:**
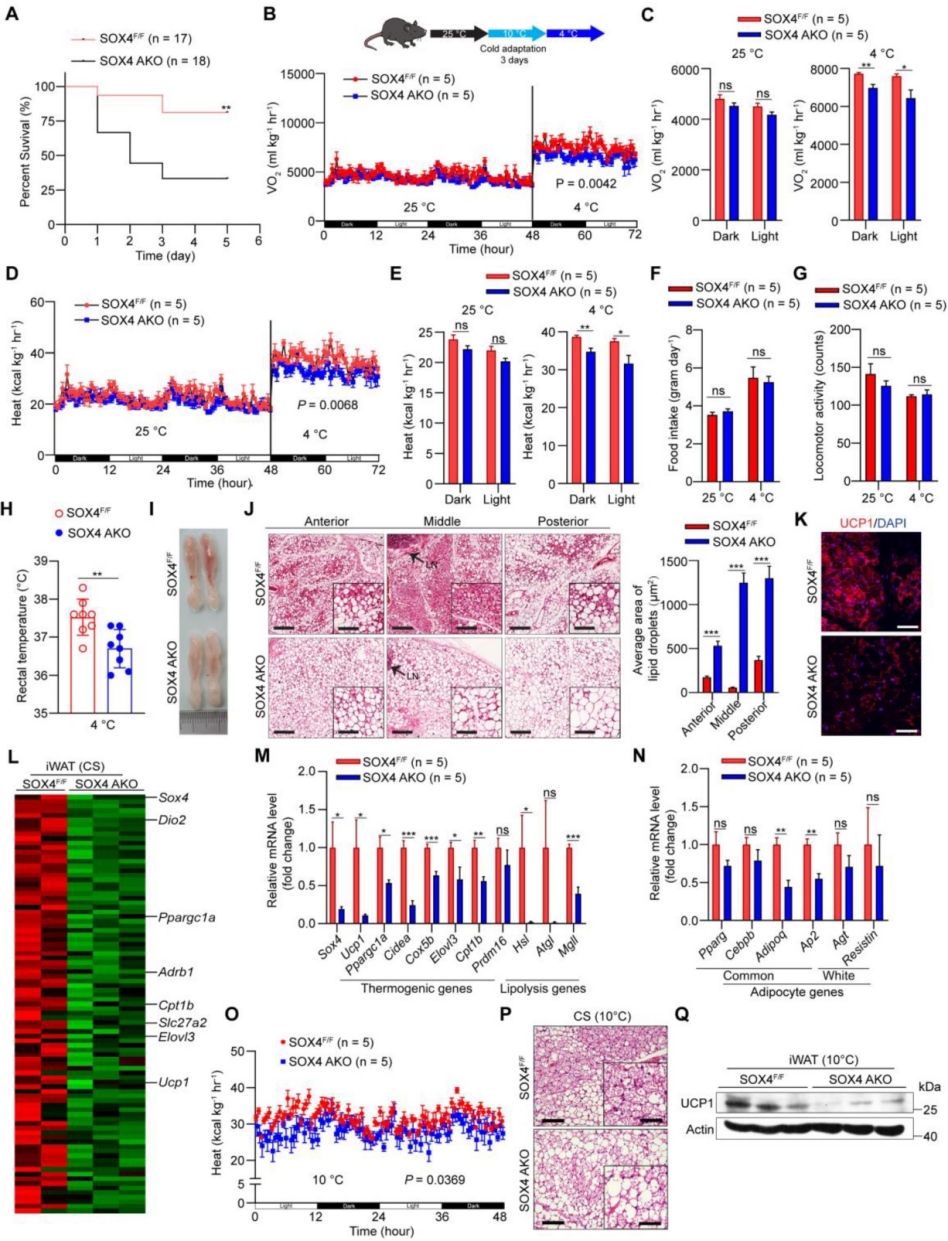
** Adipose tissue-specific SOX4 KO reduces cold tolerance, energy metabolism, and thermogenic function of beige adipocytes. (A)** SOX4^F/F^ and SOX4 AKO male mice (10-12-week) were exposed to 10 °C for one day and then to 4 °C for 5 days. Survival curves were analyzed. **(B-G)** 10-week male mice were exposed to 25 °C for 3 days, then 10 °C for 3 days and 4 °C for 3 days. Whole-body oxygen consumption (B, C), heat production (D, E), food intake (F) and locomotor activity (G) of mice at 25 °C and at 4 °C were analyzed. **(H)** SOX4^F/F^ and SOX4 AKO male mice (10-week) were exposed to 10 °C for 3 days and to 4 °C for 3 days. The core body temperature was shown.** (I-J)** Representative image and H&E staining in the iWAT in (B, D) mice. Arrowhead indicated the lymph node (LN). Scale bar, 200 µm. Insets show higher magnification, scale bar, 100 µm. The size of lipid droplets was quantified with image J (J, right). **(K)** Immunofluorescent staining of UCP1 in the middle region of iWAT in (B, D) mice. Scale bar, 50 µm. **(L-N)** SOX4^F/F^ and SOX4 AKO male mice (10-week) were exposed to 10 °C for 3 days and then to 4 °C for 3 days. iWAT was collected from each mouse, and total RNA was extracted and subjected to RNA-Seq analysis and qPCR analysis. (L) Heatmap of the RNA-Seq shows the down-regulated genes (AKO vs control) in iWAT with a cutoff of fold change ≥ 1.5 and p-value < 0.05. Thermogenic genes are indicated. (M-N) qPCR analyzed the relative mRNA levels of indicated genes in the iWAT. **(O)** SOX4^F/F^ and SOX4 AKO mice were surgically removed BAT. After recovery, mice were exposed to 10 °C for 3 days. The whole-body heat production is shown. **(P)** H&E staining in the iWAT in (O) mice. Scale bar, 100 µm. Insets show higher magnification, scale bar, 50 µm. **(Q)** Western blotting showing the expression of UCP1 protein in the iWAT in (O) mice.

To explore the effect of SOX4 on the adaptive thermogenesis of adipose tissue *in vivo*, a conditional *Sox4* allele was generated (*Sox4^lox/lox^*) by engineering loxP sites flanking exon 1 **(Figure S3A)**, named SOX4^F/F^. We next crossed SOX4^F/F^ mice to *Adiponectin*-Cre mice to generate mice with adipocyte-specific knockout of Sox4 (SOX4 AKO) and bred SOX4^F/F^ with *Ucp1*-Cre mice to produce thermogenic adipose tissue-specific SOX4 knockout (SOX4 UKO) mice. Sox4 was significantly knocked down in adipocytes of SOX4 AKO mice and in thermogenic adipose tissues of SOX4 UKO mice **(Figure S3B-D)**. Then, we performed a survival challenge experiment by exposing 10-12-week-old mice to cold environment (adaptation at 10 °C for one day and then cold stimulation at 4 °C). A total of 66% of SOX4 AKO mice, 62% of SOX4 UKO mice and 19% of SOX4^F/F^ mice died after 3 days at 4 °C** (Figure 2A, Figure S4A)**. The results reflected the obvious cold intolerance of SOX4 AKO mice and SOX4 UKO mice. The survival of mice under cold stimulation was directly related to maintaining the core body temperature.

In order to avoid the death of mice, we performed cold adaptation at 10 °C for three days and then switched to 4 °C for three days as illustrated in Figure 2B and Figure S4B. The oxygen consumption and heat production in SOX4 AKO mice and SOX4 UKO mice were significantly lower than those of SOX4^F/F^ mice at 4 °C but not at 25 °C **(Figure 2B-E; Figure S4B-E)**, while food intake and locomotor activity did not change **(Figure 2F-G, Figure S4F-G)**. Furthermore, the rectal temperatures in SOX4 AKO mice and SOX4 UKO mice were significantly lower than that of SOX4^F/F^ littermates **(Figure 2H, Figure S4H)**.

Beige adipocytes, developed form WAT postnatally in response to chronic cold exposure, are critical for non-shivering thermogenesis and maintenance of body temperature in mammals 24, 32, 33. Next, we characterized the formation of cold-induced beige fat in these mice. Prolonged cold exposure stimulated the formation of beige adipocytes with 'brown-like' and multilocular lipid droplets in the iWAT of SOX4^F/F^ mice. However, cold-induced beige fat biogenesis was strikingly impaired in the iWAT of SOX4 AKO mice and SOX4 UKO mice **(Figure 2I-J, Figure S4I-J)**. Also, immunofluorescence showed reduced expression of UCP1 in iWAT of SOX4 AKO and SOX4 UKO mice **(Figure 2K, Figure S4K)**. Next, RNA-seq analysis showed that the genes down-regulated in the iWAT of SOX4 AKO mice contained many thermogenesis-related genes under cold stimulation. Specifically, peroxisome proliferator-activated receptor-γ coactivator 1-α (*Pgc1a*), solute carrier family 27 member 2 (*Slc27a2*), carnitine palmitoyltransferase 1b (*Cpt1b*), elongation of very long-chain fatty acids-like 3 (*Elovl3*) and *Ucp1* were significantly downregulated by SOX4 KO **(Figure 2L)**. qPCR analysis further verified that the mRNA levels of thermogenesis-related genes in iWAT of SOX4 AKO mice and SOX4 UKO mice were generally down-regulated relative to SOX4^F/F^ mice **(Figure 2M, Figure S4L)**, while the common adipocyte genes *Adiponectin* and *Ap2* were slightly downregulated in SOX4 AKO mice, and the white selective genes remained unchanged **(Figure 2N, Figure S4L)**. Of note, we also analyzed the relative mRNA levels of thermogenic genes in gWAT and BAT with qPCR and found there was no significant difference in both adipose tissues between SOX4 AKO and SOX4^F/F^ mice under cold stimulation **(Figure S5A-B)**. In addition, compared to SOX4^F/F^ mice, there was no obvious morphological change in BAT of SOX4 AKO and SOX4 UKO mice with prolonged cold exposure **(Figure S5C-E)**. To further character the role of SOX4 in thermogenesis of beige adipocytes, we surgically removed the BAT from SOX4 AKO mice and SOX4^F/F^ mice. After recovery, SOX4 AKO mice exhibited decreased heat production and impaired beige fat biogenesis as well as reduced Ucp1 expression when compared with SOX4^F/F^ mice **(Figure 2O-Q)**. These results indicate that Sox4 facilitated expressions of thermogenesis-related genes and beige adipocytes formation which contribute to heat production and energy expenditure with chronic cold exposure.

### Adipose tissue-specific SOX4 KO promotes obesity and metabolic dysfunction under HFD

**Figure 3:**
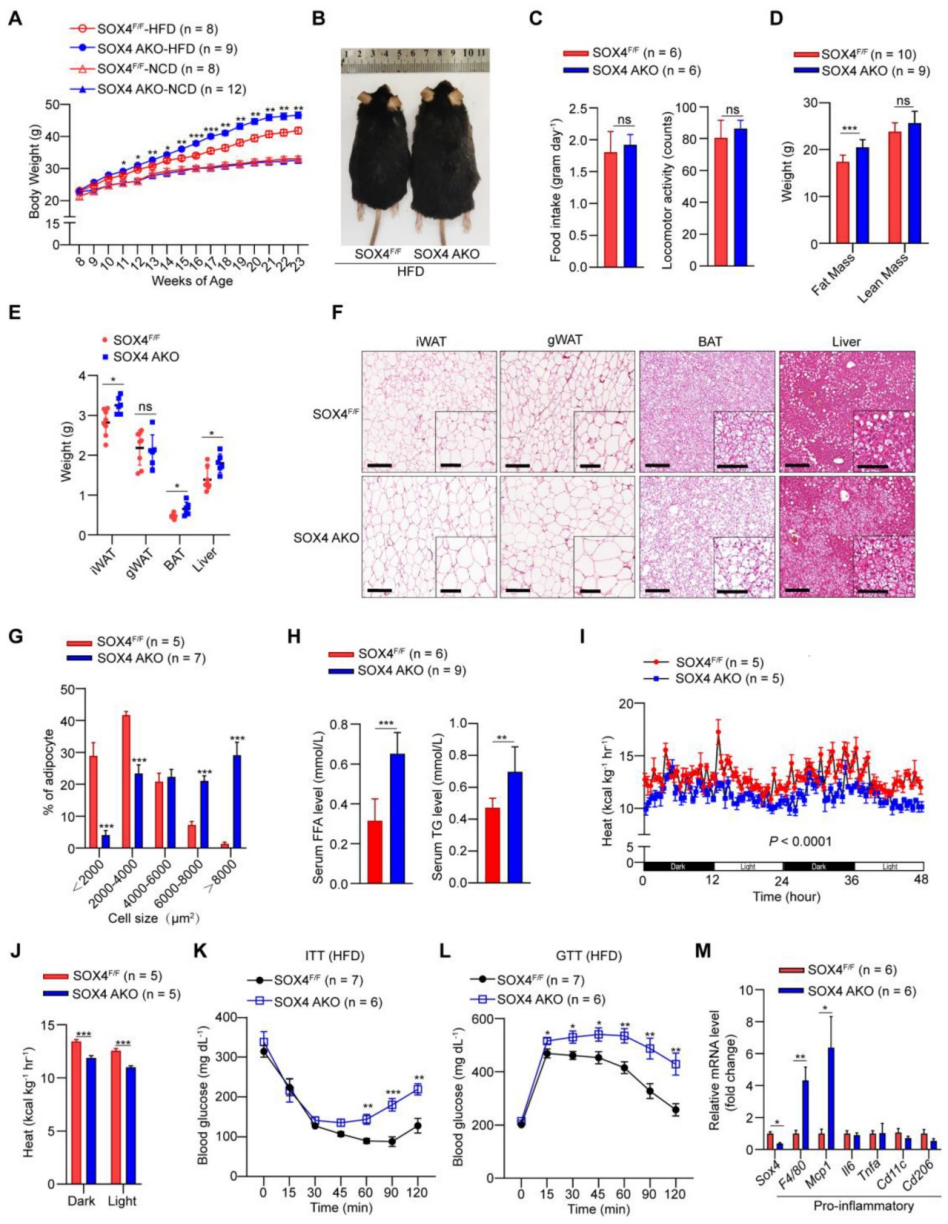
** Adipose-specific SOX4 KO promotes high fat diet-induced obesity. (A)** Growth curve of SOX4 AKO mice and control littermates fed with NCD or HFD. **(B)** A representative photo of control and SOX4 AKO mice after 15-weeks of HFD feeding. **(C)** Food intake and locomotor activity of mice in (A) after 15-weeks of HFD feeding. **(D)** The average fat and lean masses of control and SOX4 AKO mice after 15-weeks of HFD feeding. **(E)** Weights of iWAT, gWAT, BAT, and liver in control and SOX4 AKO mice after 15-weeks of HFD feeding. **(F)** Representative H&E staining of iWAT, gWAT, BAT, and liver from control and SOX4 AKO mice after 15-weeks of HFD feeding. Scale bar, 200 µm. Insets show higher magnification, scale bar, 100 µm.** (G)** Adipocyte sizes of iWAT were estimated from the H&E staining results in (F) using ImageJ. The percentage of cells with the indicated sizes was shown. **(H)** Serum levels of free fatty acid (FFA), triacylglycerol (TG) in control and SOX4 AKO mice after 15-weeks of HFD feeding. **(I, J)** Heat production of control and SOX4 AKO mice after 15-weeks of HFD. **(K, L)** insulin tolerance test (K) (i.p. 1.0 U/kg) and Glucose tolerance test (L) (i.p. 1.5 g/kg) of control and SOX4 AKO mice after 15-weeks of HFD feeding. **(M)** qPCR analysis of mRNA expression of pro-inflammatory genes in the iWAT of control and SOX4 AKO mice after 15-weeks of HFD feeding.

Since SOX4 is necessary for thermogenic gene expression and thermogenic function of beige adipocytes and regulates energy consumption in cold stimulation, we further examined the effect of SOX4 on a high-fat diet (HFD) induced obesity. We first analyzed the body weight of SOX4 AKO and SOX4^F/F^ mice fed with a normal chow diet (NCD) or HFD. SOX4 AKO and SOX4^F/F^ mice on NCD had similar body weight gain at 23 weeks **(Figure 3A)**. In contrast, with HFD for 15 weeks (age 23 weeks), the SOX4 AKO mice were fatter and gained weight faster than SOX4^F/F^ mice; SOX4 AKO mice gained 23.41g while the SOX4^F/F^ mice only gained 18.83g **(Figure 3A-B)**. Yet, there were no differences in food intake and locomotor activity between the genotypes **(Figure 3C)**. The greater body-weight gain in SOX4 AKO mice was due to increased fat mass **(Figure 3D)** and the weight of iWAT, BAT, and liver **(Figure 3E)** under HFD conditions.

Histological analysis further showed that SOX4 AKO mice fed with HFD had markedly increased lipid contents in iWAT, BAT, gWAT, and liver **(Figure 3F)**, although their gWAT weight did not differ. In particular, the adipocyte size of iWAT was significantly increased, indicating that adipose tissue-specific SOX4 KO causes iWAT cell hypertrophy. Consistently, statistical analyses showed significantly increased cell size of iWAT in SOX4 AKO mice fed with HFD **(Figure 3G)**. As expected, SOX4 AKO mice had elevated serum free fatty acid (FFA) and triglyceride (TG) levels **(Figure 3H)**, as well as lower heat production compared with SOX4^F/F^ mice **(Figure 3I-J)**. In addition, SOX4 AKO mice developed more severe insulin resistance **(Figure 3K)** and glucose intolerance **(Figure 3L)** with HFD.

We next examined the extent to which SOX4 loss alters adipose tissue inflammation. We found that, in association with their relative obesity, the iWAT of SOX4 AKO mice expressed significantly higher levels of pro-inflammatory genes *F4/80* and *Mcp1* vs. SOX4^F/F^ mice **(Figure 3M)**.

We further explored the role of SOX4 in homeostasis under NCD and room temperature. Although there was no significant difference in the morphology of iWAT, BAT, and gWAT between SOX4 AKO and SOX4^F/F^ mice **(Figure S6A)**, the mRNA level of *Ucp1* in iWAT of SOX4 AKO mice was slightly down-regulated, but not in BAT **(Figure S6B-C)**. And the relative mRNA levels of lipolysis, liposynthesis and common adipocyte genes have no significant changes in iWAT and BAT of these two genotypes **(Figure S6D-E)**. Additionally, there was no difference in body fat content and the weight of isolated adipose tissues and livers between the two genotypes **(Figure S6F-G)**. However, when measuring glucose tolerance and insulin resistance, we found that SOX4 AKO mice exhibited significant insulin resistance relative to SOX4^F/F^ mice **(Figure S6H)**, with no significant difference in glucose tolerance **(Figure S6I)**. This also further illustrates the role of SOX4 in regulating metabolic homeostasis.

### SOX4 is required for beige adipocytes development

In the previous mouse model, we observed that adipose tissue-specific SOX4 KO significantly inhibited thermogenic gene expression and thermogenic function of beige adipocytes under prolonged cold stimulation **(Figure 2)**. Thus, we further explored whether SOX4 affects the differentiation of beige adipocytes *in vitro*. We isolated SVF cells of iWAT from WT mice and immortalized them by inserting a large T antigen gene. In **Figure S7A,** beige adipocyte differentiation *in vitro* was established as described 12. mRNA levels of thermogenic genes and common adipocyte genes in differentiated adipocytes were significantly upregulated, and protein levels of UCP1 in differentiated adipocytes were obviously increased **(Figure S7B-C)**, suggesting that adipocytes differentiated from preadipocytes of iWAT SVFs were beige-like adipocytes. Subsequently, the immortalized preadipocytes were infected with lentivirus expressing scrambled or SOX4 shRNA, and then exposed to the induction media to induce beige adipocytes differentiation. At day 6 of differentiation (D6), knockdown of Sox4 caused a significant reduction in lipid content of beige adipocytes **(Figure S7D-E)**, as demonstrated in more detail by Nile red staining and bright view **(Figure S7F)**.

Next, we used RNA-seq to analyze the transcriptome of beige adipocytes (D6) with knockdown of Ctrl and *Sox4* in immortalized preadipocytes cells. Genes involved in the thermogenesis-related signaling pathways, including TCA cycle, oxidative phosphorylation, and lipolysis, were significantly down-regulated after SOX4 knockdown **(Figure S7G)**. qPCR analysis further verified that knockdown of SOX4 drastically decreased the expression of thermogenesis-related genes such as *Ucp1*, *Ppargc1α,* and *Prdm16* **(Figure S7H)**. These results show that SOX4 is required to express a thermogenic gene program in the development of beige adipocytes. Moreover, reduction of SOX4 was associated with a large decrease in mitochondrial respiration and oxygen consumption rates **(Figure S7I)**.

Based on the importance of SOX4 in the development of beige adipocytes *in vitro*, we used *Pdgfrα*-Cre and SOX4^F/F^ to cultivate a mouse model with beige preadipocytes SOX4 KO for further validation. We compared the iWAT phenotype of *Pdgfrα*-Cre: SOX4^F/F^ and SOX4^F/F^ mice after prolonged cold exposure, and found that fewer beige adipocytes with 'brown-like' and multilocular lipid droplets were generated in the iWAT of *Pdgfrα*-Cre: SOX4^F/F^ mice compared to SOX4^F/F^ mice, while the BAT of the two groups had no significant difference **(Figure S8A-B)**. qPCR analysis showed that thermogenesis-related genes in iWAT of *Pdgfrα*-Cre: SOX4^F/F^ mice were significantly reduced, while the white selective genes were significantly up-regulated, and there was no difference in general adipocyte genes (**Figure S8C-D**). Meanwhile, the expression of thermogenic genes exhibited no significant difference in BAT of both genotypes** (Figure S8E)**.

Next, we asked whether preadipocyte-specific-overexpression of Sox4 promotes the development of beige adipocytes in the condition of chronic cold exposure. The results showed that Pref1-Sox4 mice produced more beige adipocytes and heat, and expressed higher levels of thermogenic genes in iWAT compared to WT mice with chronic cold exposure, while there was no obvious change in BAT **(Figure S8F-L)**.

Together, these results demonstrated that SOX4 is required for beige fat development.

### SOX4 regulates the thermogenic function of mature beige adipocyte

**Figure 4:**
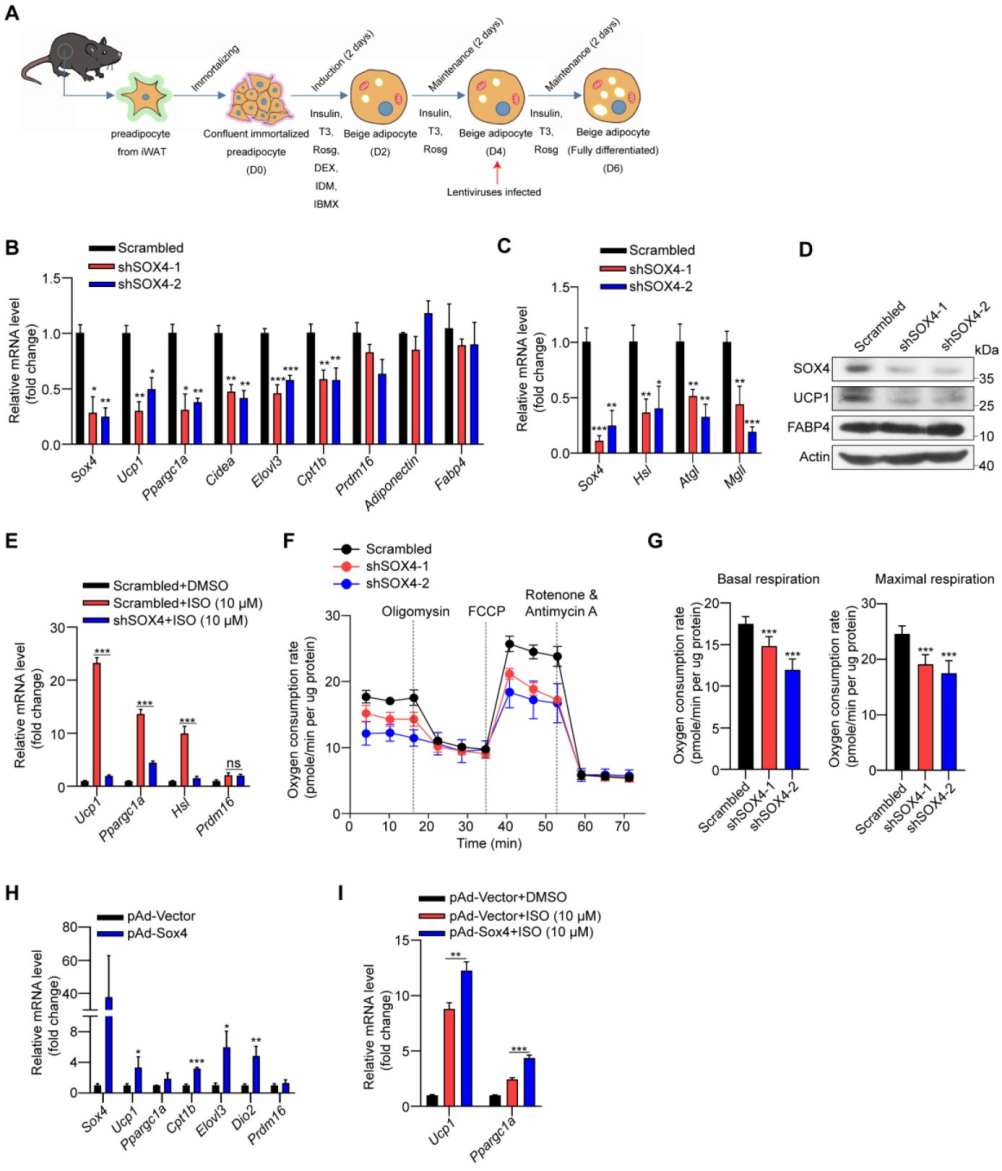
** SOX4 regulates the thermogenic function of mature beige adipocyte. (A-D)** Schematic illustration of differentiation of beige adipocytes *in vitro* (A). On day 4, cells were infected with lentivirus expressing Scrambled or shSox4. On day 6, cells were collected for qPCR (B-C) and Western blot (D) analyses.** (E)** Scrambled or shSox4 beige adipocytes (day 6) treated with or without 10 μM isoproterenol (ISO) for 4 hr. The relative mRNA levels of indicated genes were shown. **(F, G)** Oxygen consumption of Scrambled or shSox4 beige adipocytes (day 6) were analyzed. **(H)** Immortalized preadipocytes were differentiated and infected with Vector or Sox4-expression adenovirus at day 4. On day 6, cells were harvest for qPCR analyses. The relative mRNA levels of indicated genes were shown.** (I)** Immortalized preadipocyte were differentiated and infected with Vector or Sox4-expression adenovirus at day 4. On day 6, beige adipocytes were treated with or without 10 μM ISO for 4 hr. Real-time qPCR analysis of the indicated genes was performed.

Next, we explored the role of SOX4 in regulating the thermogenesis of differentiated beige fat *in vitro*. We knockdown *Sox4* on day 4 in the late stage of beige fat development **(Figure 4A)**. On day 6, differentiated beige adipocytes from immortalized preadipocyte cells were harvested. Relative mRNA and protein levels were analyzed. Knockdown of Sox4 reduced mRNA levels of thermogenesis-related genes, including thermogenic and lipolytic genes, and had no effect on adipocytes-common genes **(Figure 4B-C)**. Consistently, the protein levels of UCP1 were significantly reduced in Sox4 KD mature beige adipocytes **(Figure 4D)**. Further, decreased *Sox4* in mature beige adipocytes also weakened isoproterenol-induced the expression of thermogenic genes *Ucp1, Ppargc1α,* and lipolytic gene *Hsl* **(Figure 4E)**. Moreover, deprivation of *Sox4* in mature beige adipocytes reduced mitochondrial respiration and oxygen consumption rates **(Figure 4F-G)**.

We also infected the differentiated beige adipocytes (D4) with adenovirus carrying the *Sox4* gene to overexpress SOX4. On day 6, gene expressions were analyzed and showed that overexpression of SOX4 induced the up-regulation of thermogenesis-related gene expression **(Figure 4H)**. With isoproterenol treatment, the expression of *Ucp1* and *Ppargc1α* could be further enhanced by overexpression of SOX4 **(Figure 4I)**. The above results show that SOX4 promotes the thermogenesis of mature beige adipocytes.

### SOX4 binds to PPARγ and coactivates its transcriptional function

**Figure 5:**
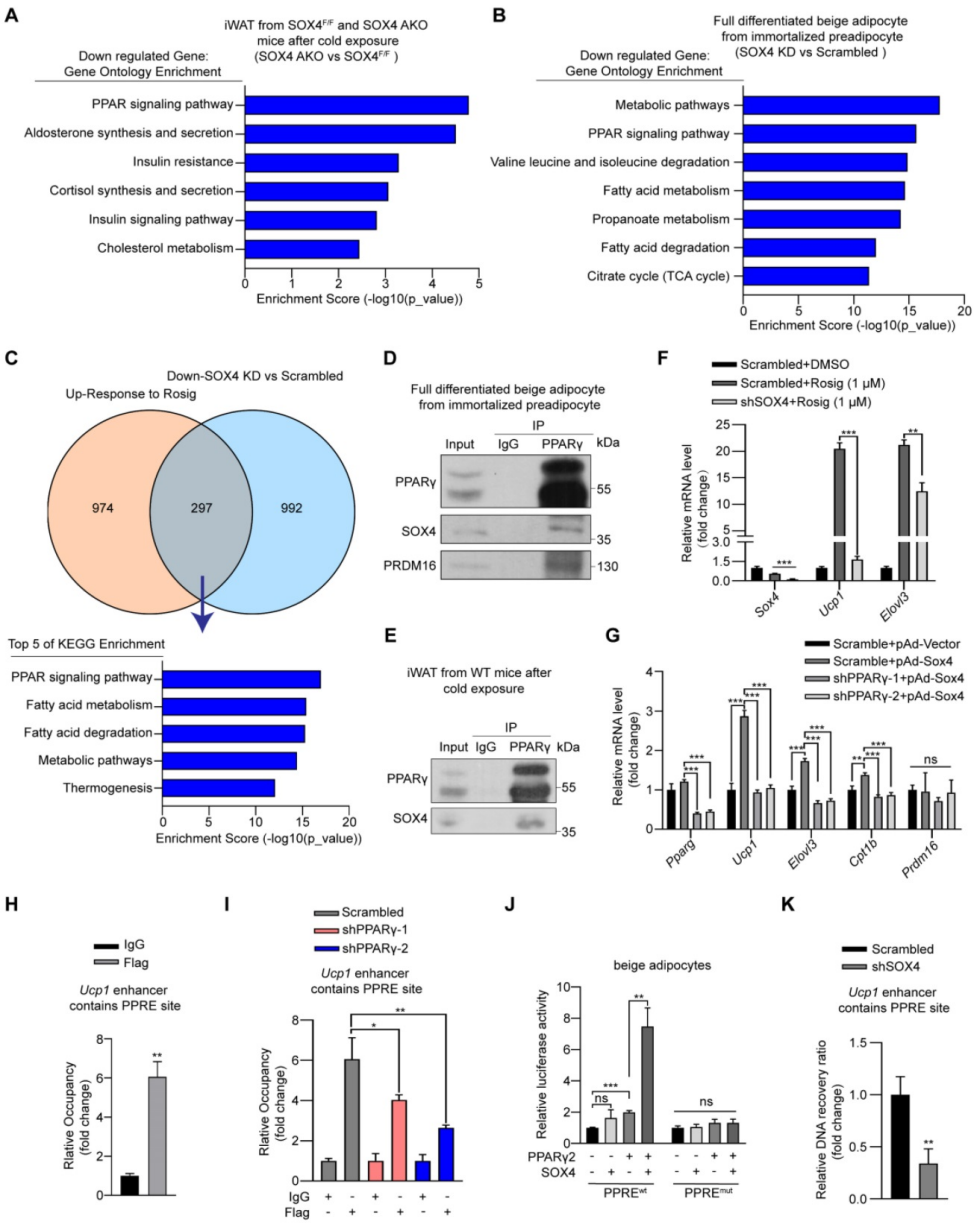
** SOX4 interacts with PPARγ2 and regulates the transcriptional activation of thermogenic genes. (A)** Genes down-regulated (SOX4 AKO vs. SOX4^F/F^ mice) in RNA-seq data from Figure 2L were subjected to gene ontology analysis. **(B-C)** Immortalized preadipocytes were subjected to beige adipocyte differentiation *in vitro*. On day 4, cells were infected with lentivirus expressing Scramble or shSox4. On day 6, cells were collected and total RNA was extracted for RNA-Seq analysis. The genes down-regulated by SOX4 knockdown were subjected to gene ontology analysis (B). Overlap (297) of genes down-regulated (992) by SOX4 knockdown in (B) and genes up-regulated (974) in Rosig-treated classic BAT (C, top, GSE144490). The overlapping genes were subjected to gene ontology analysis (C, bottom). **(D-E)** Beige adipocytes differentiated from immortalized preadipocyte (D) and iWATs of C57BL/6 mice exposed to 10 ℃ for 1 day and 4 ℃ for 1 week (E) were lysed and subjected to immunoprecipitation using IgG or anti-PPARγ antibody. Input and pellet fractions were analyzed by western blot using indicated antibodies. **(F)** Beige adipocytes differentiated from immortalized preadipocytes were infected with scrambled or shSOX4 lentiviruses on day 4. On day 6, cells were treated with or without rosig (1 μM) for 5 hr. qPCR analysis of the indicated genes were shown. The results are from 3 independent experiments. **(G)** Differentiated beige adipocytes were infected with scrambled or shPPARγ lentiviruses on day 4. On day 6, cells were infected with vector or SOX4-expression adenovirus (pAd-Sox4) treatment. 24 hr later, cells were harvested and subjected to qPCR analysis. The relative mRNA levels of indicated genes were shown. **(H)** Differentiated beige adipocytes were transfected with Flag-Sox4 on day 6. 2 days later, cells were harvested for ChIP analysis by using IgG or Flag antibody. The ChIPed DNAs were examined by qPCR for *Ucp1* enhancer containing PPRE site. **(I)** Scramble and shPPARγ beige adipocytes (day 6) were transfected with Flag-Sox4 and 2 days later subjected to ChIP assay by using IgG or anti-Flag antibody. The ChIPed DNAs were examined by qPCR for *Ucp1* enhancer containing PPRE site.** (J)** The *Ucp1* enhancer containing PPRE site or mutated PPRE site was cloned into pGL4.26-basic vector and co-transfected into mature beige adipocytes together with β-gal in the presence or absence of PPARγ2 or SOX4 expression plasmid. After 48 hr, cells were harvested and the luciferase activity was measured. β-gal activity was used to normalize for transfection efficiency. **(K)** Differentiated beige adipocytes were infected with scrambled or shSOX4 lentiviruses and subjected to FAIRE assay 2 days later. The enriched DNAs were examined by qPCR for *Ucp1* enhancer containing PPRE site.

The above results suggested that SOX4 has a key role in maintaining the thermogenic function of beige adipocytes. To further uncover its mechanism, we analyzed the transcriptome data of iWAT in SOX4 AKO and SOX4^F/F^ mice under cold stimulation **(Figure 5A)** and transcriptome data of scrambled and Sox4 knockdown beige adipocytes (D6). Signal pathway analysis of downregulated genes caused by Sox4 deletion in cells and cold stimulated iWAT tissues of mice revealed that these down-regulated genes were most strongly associated with PPAR signaling **(Figure 5A-B)**. Moreover, in the GEO database (GSE144490), the up-regulated genes after treatment of adipose tissue with rosiglitazone, a PPARγ agonist, showed 297 genes overlapping with the down-regulated genes caused by knockdown of *Sox4* in immortalized preadipocytes cells induced to differentiate into mature beige adipocytes (D6) **(Figure 5C)**. Signal pathway analysis of these 297 genes showed that these genes were mainly enriched in the PPAR signaling pathway and genes related to thermogenesis **(Figure 5C)**. This indicated that SOX4 might participate in the PPAR signaling pathway and regulate thermogenesis.

Given the central role of PPARγ in adipogenesis, we investigated whether SOX4 interacts with PPARγ to regulate the transcriptional activation of thermogenic genes. To address this question, we first performed Co-IP using HEK293T cells transfected with FLAG-tagged Sox4 and HA-tagged Pparγ2 alone or in combination. The results showed that SOX4 interacted with PPARγ2 **(Figure S9A-B)**. Here we found that although the molecular weight of endogenous SOX4 is about 37 kDa, the protein produced by the exogenous SOX4 expression plasmid is 70 kDa. We speculate that SOX4 may form a homodimer. We then confirmed the interaction between endogenous PPARγ and SOX4 using lysates of mature beige adipocytes differentiated from immortalized preadipocytes as well as iWAT lysates from cold-exposed WT mice. The lysates were immunoprecipitated with anti-PPARγ antibody followed by immunoblotted with anti-PPARγ or SOX4 antibody. The results showed SOX4 could be co-precipitated with PPARγ **(Figure 5D-E)**. In addition, immunofluorescence assay showed SOX4 colocalized with PPARγ2 in nucleus of beige adipocytes **(Figure S9C)**.

In order to further study whether SOX4 affects PPARγ activity, we introduced synthetic PPARγ agonist rosiglitazone (rosig). First, we tested whether *Sox4* depletion affected the response of beige adipocytes to rosiglitazone. We treated fully differentiated adipocytes with scrambled and shSOX4 with DMSO or rosig and detected the mRNA levels of *Ucp1* and *Elovl3*. Rosig significantly induced the expression of *Ucp1* and *Elovl3* in scrambled adipocytes but significantly weakened this induction in Sox4 KD adipocytes **(Figure 5F)**. We further found that adenovirus overexpression of SOX4 in differentiated beige adipocytes significantly increased the expression of rosig-induced thermogenesis-related genes **(Figure S9D)**. The above results indicate that SOX4 is required for rosig-mediated thermogenic gene expression.

To test whether SOX4 can directly activate the transcription of thermogenic genes in the absence of PPARγ, we knocked down PPARγ in mature beige adipocytes and found that the mRNA levels of thermogenic genes increased by SOX4 overexpression was significantly reduced **(Figure 5G)**. Next, we overexpressed FLAG-SOX4 in mature beige adipocytes induced by immortalized preadipocytes and performed Chip-qPCR. As showed in** Figure 5H**, FLAG-SOX4 could bind to PPAR-responsive element (PPRE) site on the enhancer of *Ucp1*. Moreover, knockdown of PPARγ significantly suppressed the SOX4 binding to the PPRE site on the enhancer of *Ucp1* **(Figure 5I)***.* Further, luciferase assays showed that the simultaneous expression of SOX4 and PPARγ significantly enhanced the transcription of luciferase by the *Ucp1* enhancer containing the PPRE site compared with the single expression of PPARγ in mature beige adipocyte or HEK293T cells **(Figure 5J, Figure S9E)**. However, this enhancement effect was completely blocked by mutating PPRE site **(Figure 5J, Figure S9E)**. SOX4 is known to regulate chromatin structure 34-36 and remodeling of chromatin also contributes to thermogenic activation 37-39. We thus explored whether SOX4 can facilitate chromatin openness of *UCP1*. We performed formaldehyde-assisted isolation of regulatory elements (FAIRE)-qPCR analysis and found knockdown of SOX4 significantly inhibited the openness of the PPRE site at -2.5 kb of Ucp1 enhancer **(Figure 5K)**. These indicated that SOX4 could regulate chromatin accessibility of thermogenic genes and activate the transcription in a PPARγ-dependent manner.

### SOX4 promotes the binding of PPARγ and PRDM16

**Figure 6:**
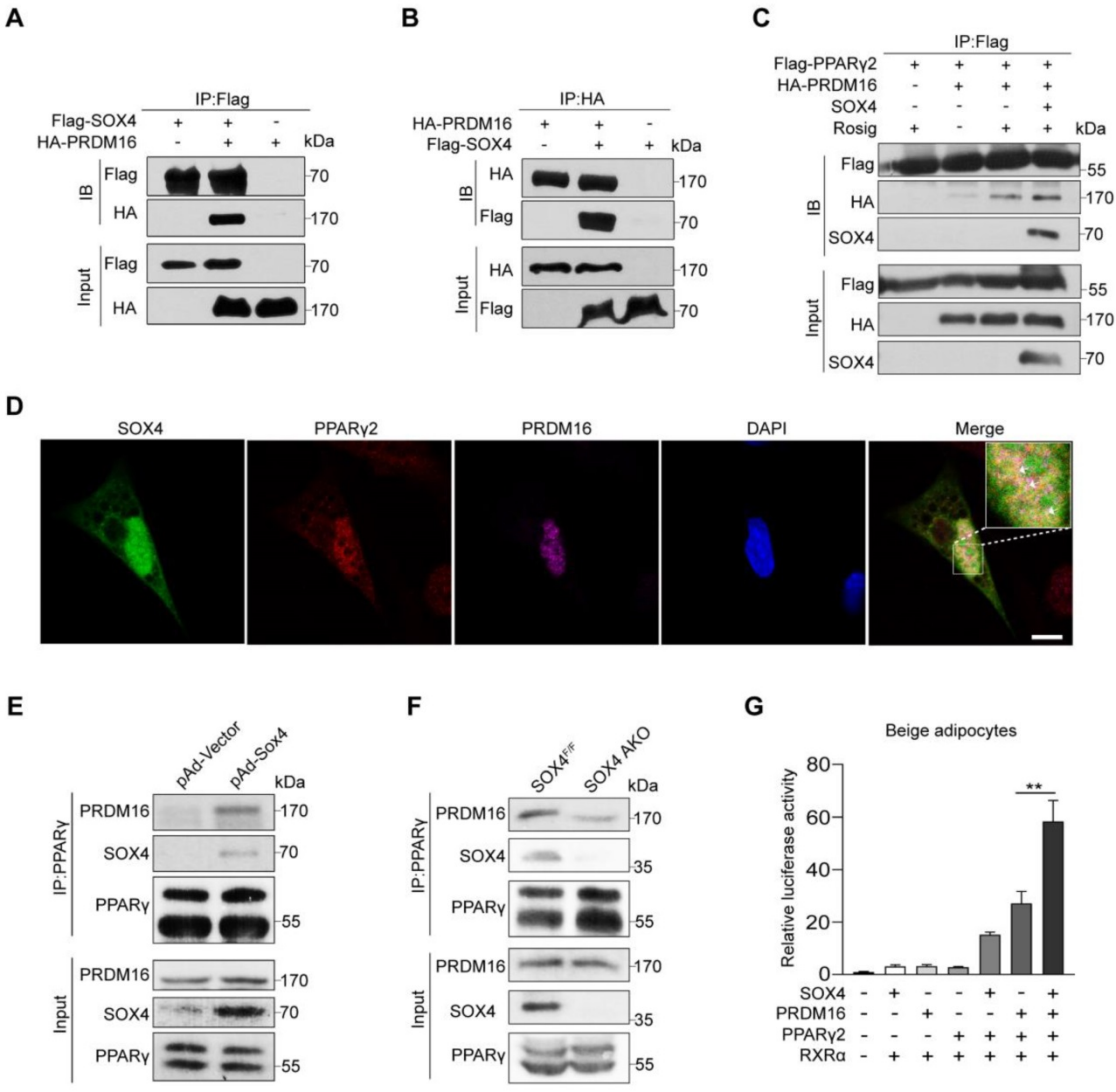
** SOX4 promotes the binding of PPARγ and PRDM16**. **(A, B)** HEK293T were transfected with Flag-SOX4 and HA-PRDM16 as indicated. 48 hr after transfection, cells were lysed and subjected into immunoprecipitation with anti-Flag (A) or anti-HA (B) antibody followed by Western blotting. **(C)** HEK293T cells transfected with Flag-PPARγ2, SOX4, and HA-Prdm16 as indicated were treated with or without 1 μM Rosig for 4 hr. Immunoprecipitation were performed as in (A). **(D)** Immunofluorescence analysis showed SOX4 was colocalized with PPARγ2 and PRDM16 in the nucleus of mature beige adipocyte (D6). Scale bar, 10 µm. **(E)** Immortalized preadipocyte were differentiated and infected with Vector or Sox4-expression adenovirus at day 4. Mature beige adipocytes (D6) were lysed, subjected into immunoprecipitation with anti-PPARγ antibody and immunoblotted with antibodies as indicated. **(F)** Control and SOX4 AKO mice were exposed to 10 °C for 3 days and then 4 °C for 3 days. iWAT were isolated and lysed. Immunoprecipitation and immunoblotting were performed as in (E). **(G)** Fragments of 3 tandem copies of a PPARγ response element fused to a luciferase reporter vector were co-transfected into mature beige adipocytes together with β-gal, PPARγ2, RXRα, and PRDM16 in the presence or absence of SOX4 expression plasmid. Luciferase activity was corrected for corresponding β-gal activity and normalized to control activity.

In our above study, we noticed that deletion of SOX4 in mature beige adipocytes did not affect the mRNA level of *Prdm16 in vivo* and *in vitro* (**Figure 2M** and** 4B**). Next, we speculated that SOX4 and PRDM16 might regulate the expression of genes by interaction rather than the relationship between upstream and downstream. In addition, PPARγ regulates the transcription of beige/brown genes in the form of complex, mostly with PRDM16 and PGC-1α, which is required for rosiglitazone or cold-induced activation of white to beige adipocytes. Therefore, we examined whether SOX4 is involved in forming the PPARγ-PRDM16 complex to regulate beige adipogenesis and function. We first performed Co-IP using HEK293T cells, and we detected the interaction of SOX4 with PRDM16 **(Figure 6A-B)**. It was reported that rosig increases the stability of PRDM16 and recruits PRDM16 to PPARγ target genes 27. Next, we examined the effect of SOX4 on the interaction between PPARγ and PRDM16 after rosig treatment. As shown, SOX4 significantly increased the interaction of PRDM16 with PPARγ2 in HEK293T cells **(Figure 6C)**. Immunofluorescence results also showed that SOX4 was colocalized with PPARγ2 and PRDM16 in nucleus in beige adipocytes **(Figure 6D)**. In addition, PGC-1α also acts as a transcriptional co-activator of PPARγ. We found that overexpression of SOX4 in HEK293T cells did not affect the binding of PPARγ2 and PGC-1α **(Figure S10)**.

To further test whether SOX4 enhances the formation of the PPARγ-PRDM16 complex, we overexpressed SOX4 with adenovirus in mature beige adipocytes and performed Co-IP. SOX4 enhanced the interaction of PRDM16 with PPARγ **(Figure 6E)**.

Next, we detected PRDM16 from the PPARγ immunoprecipitation of iWAT lysates in cold-stimulated SOX4 AKO and SOX4^F/F^ mice. We found that adipose tissue-specific SOX4 KO significantly reduced PRDM16 and PPARγ interaction **(Figure 6F)**. We then examined the effect of SOX4 on the transcriptional activity of the PPARγ-PRDM16 complex. In the luciferase experiment, we found that the simultaneous expression of SOX4, PPARγ2, and PRDM16 significantly enhanced the transcription of luciferase by the 3 tandem copies of a PPARγ response element compared with overexpression of PRDM16 and PPARγ2 in mature beige adipocyte **(Figure 6G)**. These results suggest that SOX4 increases the PRDM16-PPARγ interaction and activates the transcriptional of the thermogenic gene *Ucp1*.

## Discussion

Adaptive (non-shivering) thermogenesis is a process that drives the expression program of thermogenesis-related genes under environmental stimulation to generate heat, mainly focusing on UCP1. Transcription requires the synergy of sequence-specific DNA binding proteins and many cofactors. Cofactors do not bind to DNA and participate in transcriptional activation or inhibition. Some transcription factors that have a central role in the development and function of beige fat have been identified in previous studies, such as PPARγ 27, C/EBPβ 40, IRF4 41, FOXC2 14, and ZFP516 42. In addition, a large number of transcription cofactors that regulate the development and function of beige fat were identified, including suppressors p107 43, RIP140 44, and TLE3 45, and activators SIRT1 28, JMJD1A 32, JMJD3 46, PGC-1α 47, and PRDM16 16. Our study identified SOX4 as a positive transcriptional regulator of beige fat development and function. In the present of PPARγ, SOX4 could bind to PPRE site of Ucp1 enhancer, remodel and open the PPRE site. Besides, SOX4 recruits PRDM16 to PPARγ and form a transcription complex on the *Ucp1* enhancer to activate the expression. Also, previous studies found that SOX4 usually exerts a transcriptional regulation in the form of sequence-specific DNA binding during the development and progression of cancer 35.

Most published data on the regulation of PPARγ and beige fat are related to the development and the establishment of beige fat characteristics 41. Our work focused on the transcriptional basis of thermogenic function in differentiated beige adipocytes. Specifically, the *Adipoq*-Cre mice were used to target the knockout of the SOX4 gene in mature adipocytes 48, while *Ucp1*-Cre mice were used to target the knockout of the SOX4 gene after the establishment of the identity of brown or beige fat. SOX4 also functions in the development of beige fat. Knockdown of Sox4 inhibited the differentiation of beige adipocytes *in vitro* **(Figure S7E-H)**. Preadipocyte-specific SOX4 KO (Pdgfrα-Cre; SOX4^F/F^) greatly suppressed the development of beige fat induced by cold stimulation **(Figure S8A-D)**. Consistently, preadipocyte-specific SOX4 overexpression (Pref1-Sox4) enhanced beige adipocytes formation with prolonged cold stimuli **(Figure S8F-G)**.

Brown and beige adipose tissue are significant for maintaining body temperature in mice under cold stimulation. However, BAT develops earlier and begins in the embryonic stage. It is mainly important for the body temperature maintenance of newborns or the rapid response to short-term cold stimulation 4, 5, 32, 49. In adult mice, long-term cold stimulation induces the production of beige adipocytes in white adipose tissue to maintain the demand for heat production, which is conducive to the adaptive response to chronic cold exposure 32. The induction of heat generation genes in brown and beige fat during acute and chronic cold stress occurs through overlapping mechanisms, but there are many different mechanisms involved 25. Here, we mainly focused on the cold adaptation stage of mice after prolonged cold stimulation, and observed the effect of SOX4 KO on the expression of thermogenesis-related genes and the thermogenic function in beige fat, but not in BAT, which may be that chronic cold stress maximizes the heat production function of brown fat. Just like the minimal effect of PRDM16 and HIF2α on BAT under long-term cold stimulation 16, 50. The role of SOX4 in regulating early development of BAT and thermogenesis with acute cold exposure will be further studied in our future work.

Synthetic PPARγ agonists such as rosiglitazone are one of the most effective inducers of beige fat differentiation programs in mouse and human adipocytes 51. Studies have shown that rosiglitazone increases the stability of PRDM16 and SIRT1-dependent deacetylation of PPARγ, which recruits PRDM16 to the PPARγ target gene and promotes beige fat development 27, 28. Our experimental data show that SOX4 significantly promotes rosiglitazone-mediated thermogenic gene expression. Therefore, it will be interesting to explore whether SOX4 is involved in regulating the stability of PRDM16 and acetylating modification of PPARγ.

Selective activation of beige fat biogenesis is accompanied by increased insulin sensitivity, reduced adipose tissue inflammation, and fibrosis, which means that the beige ability of subcutaneous WAT reflects the possibility of overall metabolic health 13. We found that SOX4 AKO increases HFD-induced lipid accumulation in iWAT, gWAT, BAT, and liver. Overexpression of SOX4 (Pref1-Sox4) suppressed HFD-induced obesity and relieved glucose intolerance as well as insulin resistance.

In summary, our study reveals that SOX4 is mainly associated with PRDM16 and PPARγ2 to form a transcription complex and activate the expression of heat production genes in beige adipocytes, regulating beige adipocytes development and thermogenic function. This regulation directly affects thermogenesis and cold tolerance in mice under prolonged cold stimulation and resists obesity under HFD. Thus, SOX4 protein may potentially become a drug target for the treatment of obesity and metabolic syndrome.

## Materials and Methods

### Mice

All the animals were housed in an environment with a temperature of 22 ± 1 ºC, relative humidity of 50 ± 1%, and a light/dark cycle of 12/12 hr, with free access to water and food. All animal studies (including the mice euthanasia procedure) were done in compliance with the regulations and guidelines of Xiamen University institutional animal care and conducted according to the AAALAC and the IACUC guidelines. Food intake was recorded before and after cold exposure or HFD feeding using a 3-day metabolic cage (Sable Systems International) with singly housed mice. Plasma FFA and TG levels were measured enzymatically using a kit from Nanjing Jiancheng Bioengineering Institute. Body temperature was measured using a rectal probe (KEW Biology).

To generate SOX4^F/F^ mice, CRISPR/Cas9 recombineering was used to integrate LoxP flanked exson1 of Sox4. Ucp1-Cre mice were kindly provided by GemPharmatech Co., Ltd. Adipoq-Cre mice were obtained from Prof. JiaHuaiHan (Xiamen University). Pdgfrα-Cre (stock no.013148) were purchased from The Jackson Laboratory. Pref1-Sox4 mice were generated at Cyagen Company by inserting full-length mouse Sox4 cDNA directly after 6 kb of the Pref1 promoter in C57BL/6 mice. All mouse strains were maintained on a C57BL/6J background. A sibling or age-matched male mice were used for all the experiments.

For cold exposure, mice were singly housed at 10 ℃ for 1 or 3 days and 4 ℃ for 3 or 7 days according to the requirements of the experiment. For HFD treatment, mice at 8 weeks old were fed a diet containing 60% fat-derived calories (TestDiet) for 15 weeks. Body weight was recorded weekly for the period of HFD treatment.

For removal of BAT was performed as previously described 52, all the mice were allowed to recover for a week at room temperature before further analyses.

### Cells

Isolation of stroma-vascular fraction (SVF) cells and mature adipocytes from iWAT were performed as previously described 53, with minor modifications. Briefly, iWATs were excised from 8-week-old male mice, minced into pieces with scissors, and then incubated with digestion buffer containing 3 mg/ml type II collagenase (C6685-1G; Sigma-Aldrich) and 0.3% BSA (Sigma-Aldrich) in HBSS (BI) in a ratio of 5 ml/g tissue at 37 ℃ for 60 mins, followed by centrifugation at 500 g for 5 min. Mature adipocytes were then collected, washed with PBS, and mixed with Trizol for RNA extraction. Pellets containing SVF cells were rinsed three times in PBS, collected by centrifugation at 500 g for 5 min, and resuspended with DMEM/High glucose (BI) supplemented with 10% fetal bovine serum (Gbico), penicillin (100 U/ml, Gibco) and streptomycin (100 mg/ml, Gbico). The SVF cells were cultured in a humidified atmosphere containing 5% CO_2_/95% air at 37 ºC. The SVF cells adhered to the wall for two hours and were washed with PBS three times to remove impurities. SVFs were immortalized using the SV40 Large T antigen (pBabe SV40 Large T antigen; Addgene) according to the cell immortalization protocol 54.

### *In vitro* Beige adipocyte differentiation

Beige adipocyte differentiation *in vitro* was carried out as previously described 12. Briefly, confluent SVF cells (designated as Day 0, D0) were cultured in the induction media containing 0.5 mM 3-isobutyl-1-methylxanthine (IBMX, Sigma), 1 uM dexamethasone (DEX, Sigma), 125 nM indomethacin (IDM, Sigma), 850 nM insulin (MCE), 1 nM 3,3',5-Triiodo-L-thyronine (T3, Sigma) and 1 μM rosiglitazone (Rosig, MCE) for 2 days (designated as Day 2, D2) and then in maintenance media containing 850 nM insulin, 1 nM T3 and 1 μM rosiglitazone every other day (designated as Day 4, D4) till Day 6 (designated as Day 6, D6). To activate thermogenic gene expression, Rosiglitazone was abandoned from the maintenance medium after four days of beige adipocyte induction, and isoproterenol (10 μM. Sigma) or rosiglitazone (1 μM, MCE) treated fully differentiated adipocytes (D6) for 4 hr before cells harvesting.

### Western Blot

Protein was extracted from tissues and cells with TRIzol reagent (Invitrogen) according to the manufacturer's instructions and supplemented with a complete protease inhibitor cocktail (Roche) and 1 mM PMSF (Sigma). The protein content was measured using BCA Protein Assay Kit (Thermo Fisher Scientific, 23228). For immunoblotting, A 120-180 μg protein was denatured in SDS sample buffer, resolved using a 10% SDS-PAGE, and transferred onto polyvinylidene fluoride (PVDF) transfer membrane (Mllipore). The membrane was then blocked with 10% blotting grade milk powder in TBST (50 mM Tris-HCl, 0.15 M NaCl, 0.1% Tween-20, pH7.4) and incubated with primary antibodies for SOX4 (Abcam, #ab70598), UCP1 (Cell Signaling, #14670), PPARγ (Proteintech, #16643-1-AP), PRDM16 (Abcam, ab106410), PGC-1α (Mllipore, AB3242), HA (Sigma-Aldrich, H6908), Flag (Sigma-Aldrich, F7425), β-actin (Sigma, A1978) at 4 ℃ overnight. Membranes were then washed and incubated with anti-rabbit-HRP for UCP1, PPARγ, PRDM16, PGC-1α, HA, and Flag and anti-mouse-HRP for SOX4, β-actin for 1h at room temperature. Enhanced chemiluminescence was analyzed using Immobilon ECL Ultra Western HRP Substrate (Mllipore, WBULS0100) and subjected to chemiluminescence imaging.

### RNA purification, Reverse Transcription, and Quantitative PCR

Total RNA was extracted from tissues and cells with TRIzol reagent (Invitrogen) according to the manufacturer's instructions. A 2-4 μg of total RNAs was converted to cDNA with Random primers/Oligo (dT)_20_VN primer mix using HiScript III RT SuperMix for qPCR (+ gDNA wiper) (Vazyme, R323). Quantitative PCR was performed in the CFX384 (BIO-RAD) with specific primers and UltraSYBR Mixture (CWBIO) according to the manufacturer's instructions. The relative abundance of mRNAs was standardized with 18s mRNA as the invariant control. All real-time qPCR reactions were carried out in triplicate. PCR primers are shown in supplemental **Table S1.**

### Lentiviral Production and Infection

For construction of knockdown lentiviral plasmids, the shRNA sequences of Sox4 and Pparγ are listed in supplemental **Table S2**, which were cloned into a pLKO.1 lentiviral vector. To generate lentivirus particles, lentiviral constructs were co-transfected with pM2D.G and psPAX2 into HEK293T cells as described 55. Media containing lentivirus was harvested, filtered, and concentrated at 48 and 72 hr post-transfection. For beige adipocytes differentiation. on day -2, 70% of confluent immortalized preadipocyte were incubated with lentivirus premixed with polybrene (10 μg/ml) for 48 hours. From day 0, cells were cultured in induction media for 2 days and maintenance media for 4 days. For beige adipocytes function research, beige adipocytes differentiated on day 4 were incubated with maintenance media containing lentivirus premixed with polybrene (10 μg/ml) for 48 hours. The beige adipocytes were harvested and used for analysis on day 6.

### Mitochondrial Function and Respiration

Mitochondrial oxygen consumption rates (OCRs) in beige adipocytes were measured using a Seahorse XFe96 Extracellular Flux Analyzer (Agilent). Briefly, immortalized-SVF cells (6×10^3^ per well) were seeded into XFe96 cell culture microplate (Agilent) and induced beige adipocyte differentiation, with lentiviral infection at day 2 or day 4 of differentiation. Cells at day 6 of differentiation were equilibrated in XF assay medium supplemented with 1 mM sodium pyruvate, 2 mM GlutaMAX-I, and 25 mM glucose for 1 h in a 37 ℃ incubator without CO_2_ before analysis. The XF96 plates were then transferred to a temperature-controlled (37 ℃) Seahorse XFe96 analyzer, followed by equilibration for 12 min and 3 assay cycles (the samples were mixed for 3 min. Then, the basal respiration rate was determined. Oligomycin (4 μM), carbonyl cyanide 4-(trifluoromethoxy) phenylhydrazone (FCCP, 2.5 μM), and rotenone/actinomycin A (3 μM each) were then sequentially added into the microplate by automatic pneumatic injection, followed by the measurement of OCR by ATP production, maximal respiration rate, and spare respiratory capacity with 3 assay cycles (mix the samples for 3 min, wait for 2 min and measure the rates for 3 min) for each compound. OCRs were recorded at the time points indicated in the figures. Total proteins from beige adipocytes of each well were extracted after OCR measurement assays using cell lysis buffer (50 mM Tris-HCl, 0.02 g/mL SDS, Ph 6.8) and quantified using BCA Protein Assay Kit (Thermo Fisher Scientific, 23228) for normalization. All compounds mentioned above were obtained from XF Cell Mito Stress test kit (Agilent). Data was analyzed using Seahorse Wave Desktop Software (Agilent).

### Immunoprecipitation

For Co-IP experiments using tagged constructs, HEK293T cells were transfected using PEI (Polysciences, 23966-1) to express FLAG-tagged protein and HA-tagged protein. Cells were lysed in IP buffer containing 20 mM Tris, pH 7.5, 150 mM NaCl, 1 mM EDTA, 1 mM EGTA, 2.5 mM sodium pyrophosphate, 1% Triton-100 supplemented with both protease inhibitors cocktail (Roche), and phosphatase inhibitors (PMSF, Sigma). Total cell lysates were incubated with indicated antibodies of affinity gel (MCE).

PPARγ immunoprecipitation was performed on beige adipocytes differentiated from immortalized-SVF cells or iWAT tissue. The total cell lysates in lysis buffer containing 20 mM Tris, pH 7.5, 150 mM NaCl, 1 mM EDTA, 1 mM EGTA, 2.5 mM sodium pyrophosphate, and 1% Triton-100 supplemented with both protease inhibitors cocktail and PMSF (Sigma) was incubated overnight with 2 μg of anti-PPARγ antibody (Proteintech, #16643-1-AP) previously cross-linked to protein A/G magnetic beads (MCE) followed by washing with lysis buffer. Immunoprecipitated PPARγ and co-immunoprecipitated SOX4 and PRDM16 were detected by Western blotting.

### Chromatin Immunoprecipitation (ChIP) Assay

Chromatin immunoprecipitation was performed using ChIP Assay Kit (Beyotime, P2078) following the manufacturer's protocol. Beige adipocytes differentiated from immortalized preadipocyte at day 6 were transfected with the Flag-Sox4 and fixed with 4% formaldehyde for 15 min at day 8, followed by 0.125 M glycine quenching for 5 min. Adipocytes were scraped and lysed in SDS lysis buffer supplemented with a complete protease inhibitor cocktail (Roche) and 1 mM PMSF (Sigma), and the DNA was fragmented by sonication to approximately 200-1000 bp. Immunoprecipitation was performed with 20 μl Flag-beads (MCE, HY-K0207) in 2ml lysate overnight at 4 ℃. Antibody-bound chromatins were washed, eluted, and reverse cross-linked. ChIPed DNA was extracted by phenol/chloroform, and ethanol precipitated. The immunoprecipitated DNA was quantified by qPCR using CFX384 (BIO-RAD). The PCR primers are shown in supplemental **Table S3**.

### Formaldehyde-assisted Isolation of Regulatory Elements (FAIRE) Assay

Immortalized preadipocyte were subjected to beige adipocyte differentiation for 4 days and infected with Scramble or shSOX4 lentivirus for 48 hr. Cells were cross-linked for 10 min at room temperature by adding formaldehyde directly to the culture medium at a final concentration of 1% (v/v). The formaldehyde was quenched by the addition of 0.125 M glycine for 5 minutes at room temperature. After three washes with cold PBS supplemented with 1 mM PMSF, samples were collected and lysed by lysis buffer (1% SDS containing 10 mM EDTA, 50 mM Tris-HCl pH 8.1, 1 mM PMSF and 1 mM cocktail) for 10 min on ice. Cell lysates were sonicated under appropriate conditions to obtain 200-1,000 bp DNA fragments. After centrifugation, the supernatant for each group was collected and incubated with RNase A for 1h at 37 ℃. Then the samples were split into two equal parts. For decrosslinking (control DNA), 10µL of proteinase K (20 mg/mL) was added and incubated at 37 ° C for 4 hr, 65 ° C for 6 hr. The non-de-crosslinked and de-crosslinked samples were purified by phenol: chloroform. The chromatin accessibility was measured by qPCR and assessed according to the calculation method reported previously 56. The primers (targeting the PPRE site at - 2.5 kb of *Ucp1* enhancer) used for FAIRE-PCR are list as below:

Forward: 5'-CACGGACACTAGGTAAGTGAAGCTTG-3';

Reverse: 5'-GAGTCTGATTTCTGCTCTTCTGGCA-3'.

### Luciferase Reporter Assays

The mouse *Ucp1* enhancer containing the PPRE site (positions -2720 to -2102 bp) or 3 tandem copies of a PPARγ response element (3 × DR1-Luciferase) 57 was inserted into the pGL4.26 vector. Differentiated beige adipocytes cells were cultured in 24-well plates and co-transfected with PPARγ plasmid (30 ng/well), RXRα plasmid (15 ng/well), SOX4 plasmid (60 ng/well), PRDM16 plasmid (60 ng/well), luciferase reporter construct (30 ng/well), and galactosidase expression vector (control reporter) (15 ng/well) using PEI (Polysciences, 23966-1). The mass of transfected plasmids was balanced with an empty vector. After 48 hr, cells were harvested, and the luciferase activity was measured. β-gal activity was used to normalize for transfection efficiency. All luciferase assay experiments were performed in triplicate.

### Oil red O staining and Nile red staining

Cells at the specified stage of differentiation were rinsed with PBS and fixed with 4% formaldehyde in PBS for 10 min. After two washes in PBS, cells were stained with oil red O at RT for 10 min. The stain was then removed, and the cells were washed twice with PBS and photographed. For Nile red staining, fixed cells were stained in a working solution (3% BSA-PBS solution, 0.2 μg/ml Nile red, 5 µg/ml DAPI) for 10 minutes at 22 ℃ in the dark. The cells were then rinsed with PBS to remove extra solution and photographed.

### Body Composition and Indirect Calorimetric Analysis

Body composition was measured in non-anesthetized mice using magnetic resonance imaging (EchoMRI). For indirect calorimetry, mice were housed individually in cages equipped with the Promethion system for monitoring indirect calorimetry, physical activity, and food and water intake. Mice were maintained under a 12-hr light/12-hr dark cycle. The concentration and flow of O_2_ and CO_2_ as well as the environmental temperature, were monitored for 36-48 hr.

### Glucose and Insulin Tolerance Tests

For glucose tolerance tests, mice on HFD for 15 weeks or NCD received an intraperitoneal injection of glucose (1.5 g/kg body weight) after 16 hours of fasting. For insulin tolerance tests, mice on HFD for 15 weeks received an intraperitoneal injection of insulin (1.0 U/kg body weight) after 6 hours of fasting; mice on NCD received an intraperitoneal injection of insulin (0.75 U/kg body weight) after 6 hours of fasting. Glucose level was measured in tail blood at 0, 15, 30, 60, 90, and 120 mins after glucose or insulin injection using a glucometer (ONETOUCH UltraEasy).

### Tissue histology and immunohistochemistry

For hematoxylin and eosin (H&E) staining, the tissues were fixed in 4% formaldehyde overnight at 4 ℃. After the dehydration procedure, tissues were embedded in paraffin and sectioned (5 µm thickness). Paraffin-embedded tissues were then deparaffinized twice in xylene and subsequently rehydrated, stained with H&E. Images were acquired using a microscope (Leica Aperio Versa 200).

For immunostaining, paraffin-embedded tissues were deparaffinized twice in xylene and subsequently rehydrated. After incubating the slides for 20 min in boiling water, the tissues were blocked in PBS containing 10% goat or donkey serum with 0.1% Tween 20 for 60 min. After washing in PBS, slides were incubated with rabbit anti-UCP-1(1: 100, A5857, ABclonal) antibody overnight at 4 ℃, followed by incubation with fluorescence conjugated the second antibody for 60 min at room temperature. Alexa Fluor 555 antibody (1:500, Thermo Fisher) was used as a second antibody for UCP1. After washing, the sections were stained with 4',6-diamidino-2-phenylindole (DAPI). Images of tissue samples were captured using the Leica TCS SP8 DLS and analyzed using the LAS X software.

For immunohistochemistry, paraffin-embedded tissues were deparaffinized twice in xylene and subsequently rehydrated. After incubating the slides for 20 min in boiling water, tissue sections were incubated with 3% H_2_O_2_ (MXB) for quenching endogenous peroxidases, permeablized by 0.1% Triton X-100, and incubated with SOX4 Ab (Mllipore, #AB10537) overnight. Subsequently, adipocytes with SOX4 Ab labeling were detected using goat anti-rabbit IgG conjugated with HRP, following the DAB chromogenesis kit (MXB) instructions.

### RNA-Seq

Total RNA was extracted using Trizol reagent (Invitrogen, Carlsbad, CA, USA) according to the manufacturer's protocol. RNA quality was assessed on an Agilent 2100 Bioanalyzer (Agilent Technologies, Palo Alto, CA, USA) and checked using RNase free agarose gel electrophoresis. After total RNA was extracted, mRNA was enriched by Oligo(dT) beads. Then the enriched mRNA was fragmented into short fragments using fragmentation buffer and reverse transcribed into cDNA with random primers. Second-strand cDNA was synthesized by DNA polymerase I, RNase H, dNTP, and buffer. Then the cDNA fragments were purified with a QiaQuick PCR extraction kit (Qiagen, Venlo, The Netherlands), end-repaired, mixed with poly(A), and ligated to Illumina sequencing adapters. The ligation products were size selected by agarose gel electrophoresis, PCR amplified and sequenced using Illumina HiSeq2500 by Gene Denovo Biotechnology Co. (Guangzhou, China).

### Statistical Analysis

Each experiment was repeated at least three times. Representative experiments are shown unless stated otherwise. Data was analyzed using the GraphPad software package. Data are presented as the means ± SD (*in vitro* experiments) or SEM (*in vivo* experiments) and were analyzed by unpaired two-tailed student t-test. P < 0.05 was considered statistically significant (*P < 0.05, **P < 0.01, ***P < 0.001; ****P < 0.0001; ns, no significance). No randomization was used, and the experimenter was blinded to genotypes.

## Supplementary Material

Supplementary figures and tables.

## Abbreviations

RNA-seq: RNA sequencing
WAT: white adipose tissue
iWAT: inguinal white adipose tissue
BAT: brown adipose tissue
qPCR: real- time fluorescence quantitative PCR
Co-IP: co-immunoprecipitation
ChIP: chromatin immunoprecipitation
SOX4: transcription factor SOX-4
PPARγ: peroxisome proliferator-activated receptor gamma
PRDM16: histone-lysine N-methyltransferase PRDM16
HFD: high-fat-diet
NCD: normal chow diet
SVF: stromal-vascular fraction
DMSO: dimethyl sulfoxide
ISO: isoproterenol
Rosig: rosiglitazone
RT: room temperature
CS: cold stimulation
GTT: glucose tolerance tests
ITT: insulin tolerance tests
pAd: Porcine adenovirus
