# Supplementary material for: SOX4 promotes beige adipocyte-mediated adaptive thermogenesis by facilitating PRDM16-PPARγ complex
Source: Theranostics. 2022 Nov 7;12(18):7699–716. doi: 10.7150/thno.77102 (PMC9706582; doi:10.7150/thno.77102)
Supplement: Supplementary file 1 — Supplementary figures and tables. [file thnov12p7699s1.pdf]

Supplementary Information

SOX4 promotes beige adipocyte-mediated adaptive thermogenesis by facilitating PRDM16-PPARγ complex

Huanming Shen[1†], Ting He[1†], Shuai Wang[1†], Lingfeng Hou[1†], Yixin Wei[1], Yunjia Liu[1], Chunli Mo[1], Zehang Zhao[1], WeiXin You[1], Huiling Guo[1,*], and Boan Li[1,2*]

Figure S1

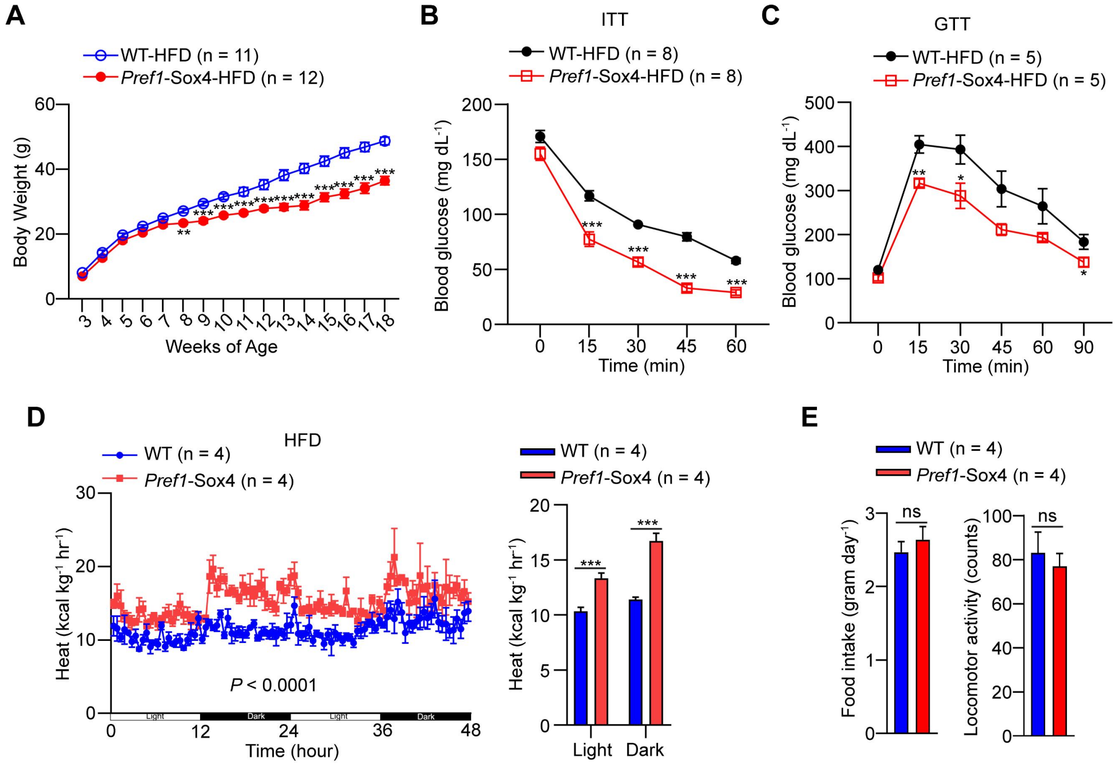

Figure S1. Under HFD, Pref1-Sox4 mice increased heat production compared with WT mice.

(A-C) Control and Pref1-Sox4 male mice were fed with HFD and housed at room temperature (25 °C). Growth curve (A), insulin tolerance test (B), glucose tolerance test (C), whole-body heat production **(D)**, food intake and locomotor activity **(E)** were analyzed.

Figure S2

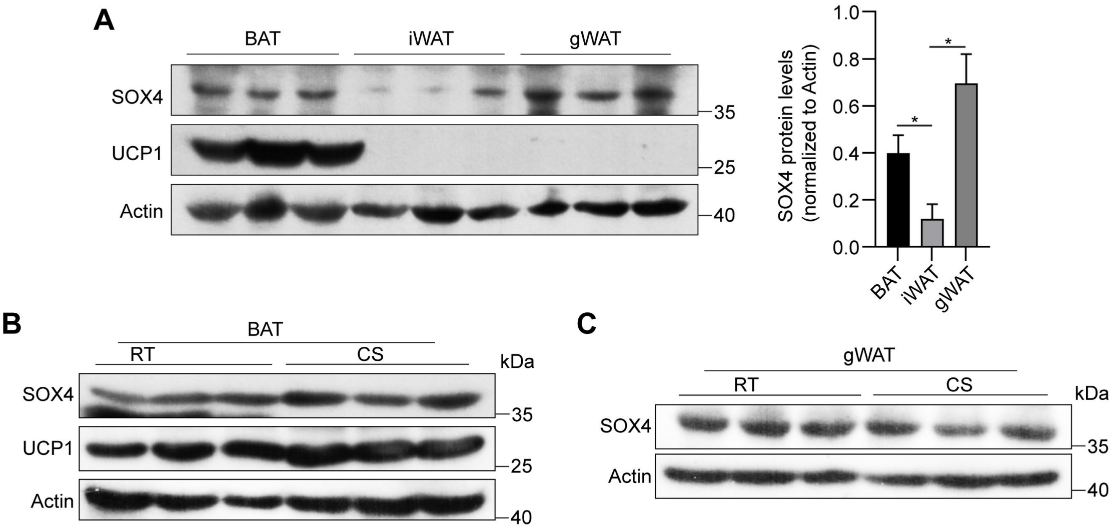

Figure S2. The expression of SOX4 in three adipose tissues under room temperature or cold stimulation.

(A) Western blot analysis of SOX4 protein in iWAT, BAT and gWAT of 10-week WT mice. Band intensity of SOX4 was quantified using Image J and normalized to that of actin. **(B, C)** 10-week male mice were housed at room temperature (RT) or exposed to 10 °C for 1 day and then 4 °C for 1 week. BAT and gWAT were isolated and subjected to Western blotting.

Figure S3

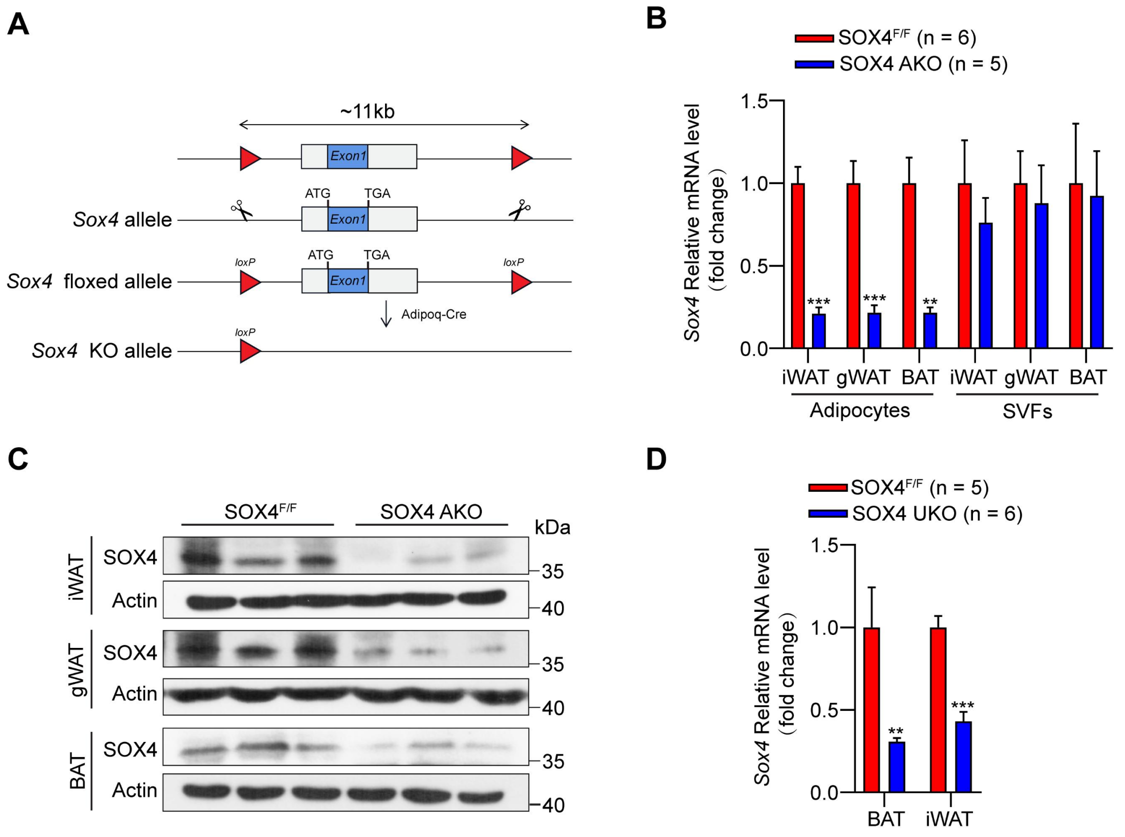

Figure S3. Construction of tissue specific SOX4 knockout mice and validation of knockout efficiency.

(A) Schematic diagram of SOX4$^{F/F}$ mouse construction. **(B)** Sox4 mRNA levels in mature adipocytes and SVFs isolated from iWAT, gWAT and BAT of 12-week control and SOX4 AKO male mice at room temperature. **(C)** 12-week control and SOX4 AKO male mice were exposed to 10 °C for 3 day and then 4 °C for 1 week. The protein levels of SOX4 in iWAT, gWAT and BAT were shown. **(D)** Sox4 mRNA levels in iWAT and BAT of 12-week control and SOX4 UKO mice at room temperature.

Figure S4

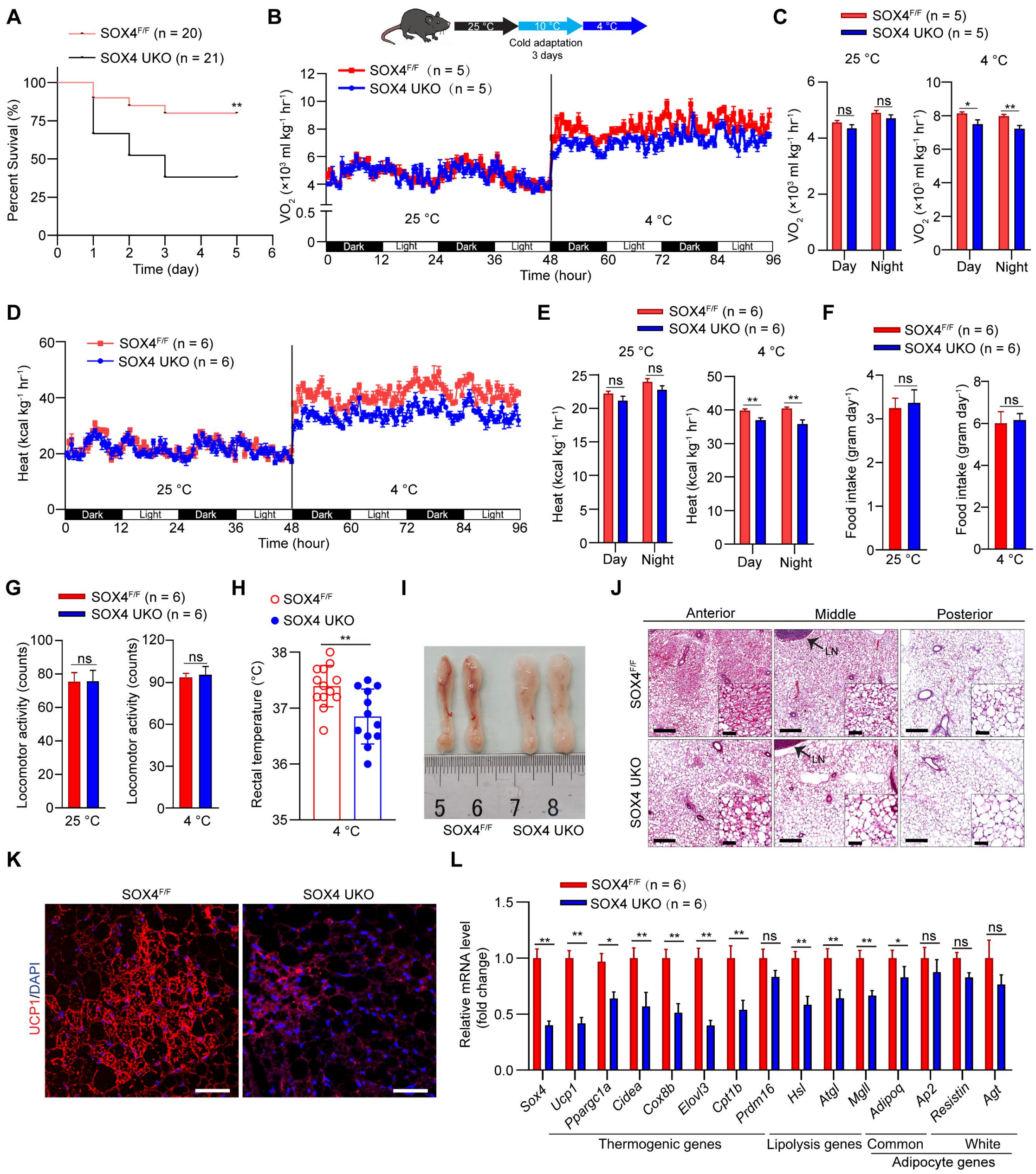

Figure S4. Thermogenic adipose tissue-specific SOX4 KO attenuates beige adipose thermogenesis, energy metabolism and body temperature maintenance.
(A) SOX4$^{F/F}$ and SOX4 UKO male mice (12-week) were exposed to 10 °C for one day and then switched to 4 °C for 5 days. Survival curves were analyzed. **(B-G)** 12-week SOX4$^{F/F}$ and SOX4 UKO male mice were exposed to 25 °C for 3 days, then 10 °C for 3 days and 4 °C for 3 days. Whole-body oxygen consumption (B, C), heat production (D, E), food intake (F) and locomotor activity (G) of mice at 25 °C and at 4 °C were analyzed. **(H)** The core body temperature of SOX4$^{F/F}$ and SOX4 UKO male mice (10-week) which were exposed to 10 °C for 3 day and to 4 °C for 3 days. **(I-J)** Representative image (I) and H&E staining (J) in the iWAT in (B, D) mice. Arrowhead indicates lymph node (LN). Scale bar, 200 μm. Insets show

higher magnification, scale bar, 50 μm. **(K)** Immunofluorescent staining of UCP1 in the middle region of iWAT in (B, D) mice. Scale bar, 50 μm. **(L)** 12-week control and SOX4 UKO male were treated as in (B). iWATs were isolated and subjected into qPCR analysis.

Figure S5

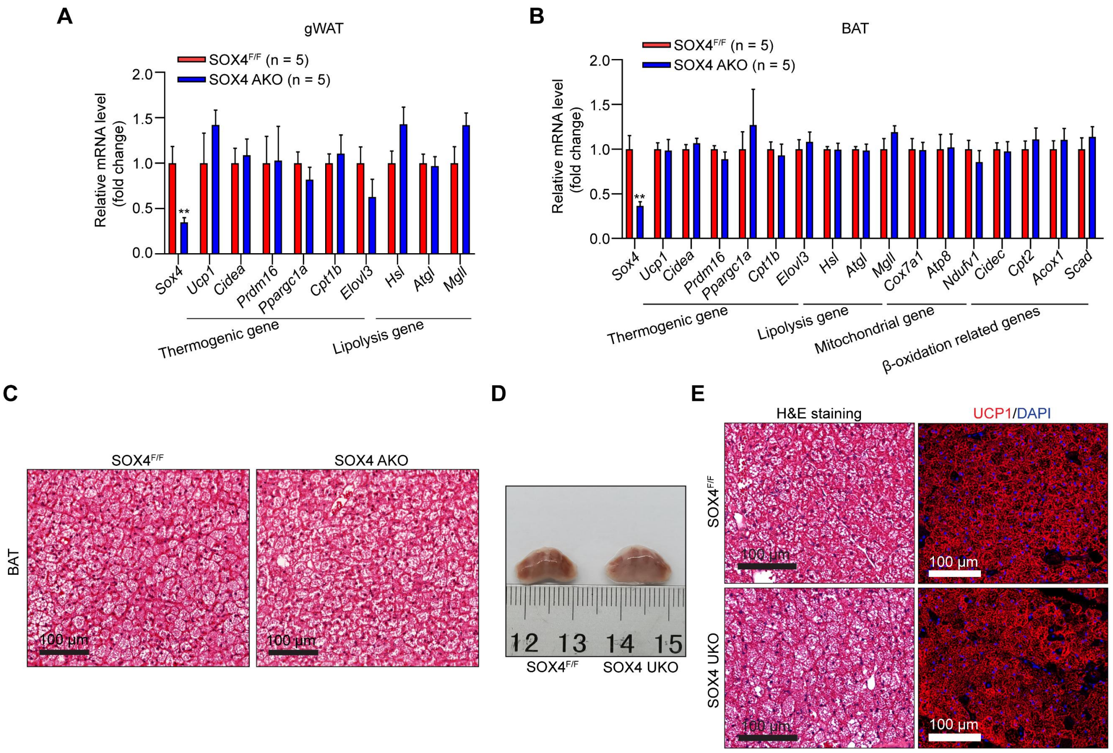

Figure S5. SOX4 KO had minor effect on BAT with prolonged cold exposure. **(A-C)** 10-week control and SOX4 AKO male mice were treated as in Figure 2B. gWAT (A) and BAT (B) were isolated and subjected to qPCR analysis. (C) Representative H&E staining of BAT. Scale bar, 100 μm. **(D-E)** 12-week control and SOX4 UKO male mice were treated as in Figure 2B. Representative image of BAT (D), H&E and UCP1 immunofluorescent staining of BAT (E) were shown. Scale bar, 100 μm.

Figure S6

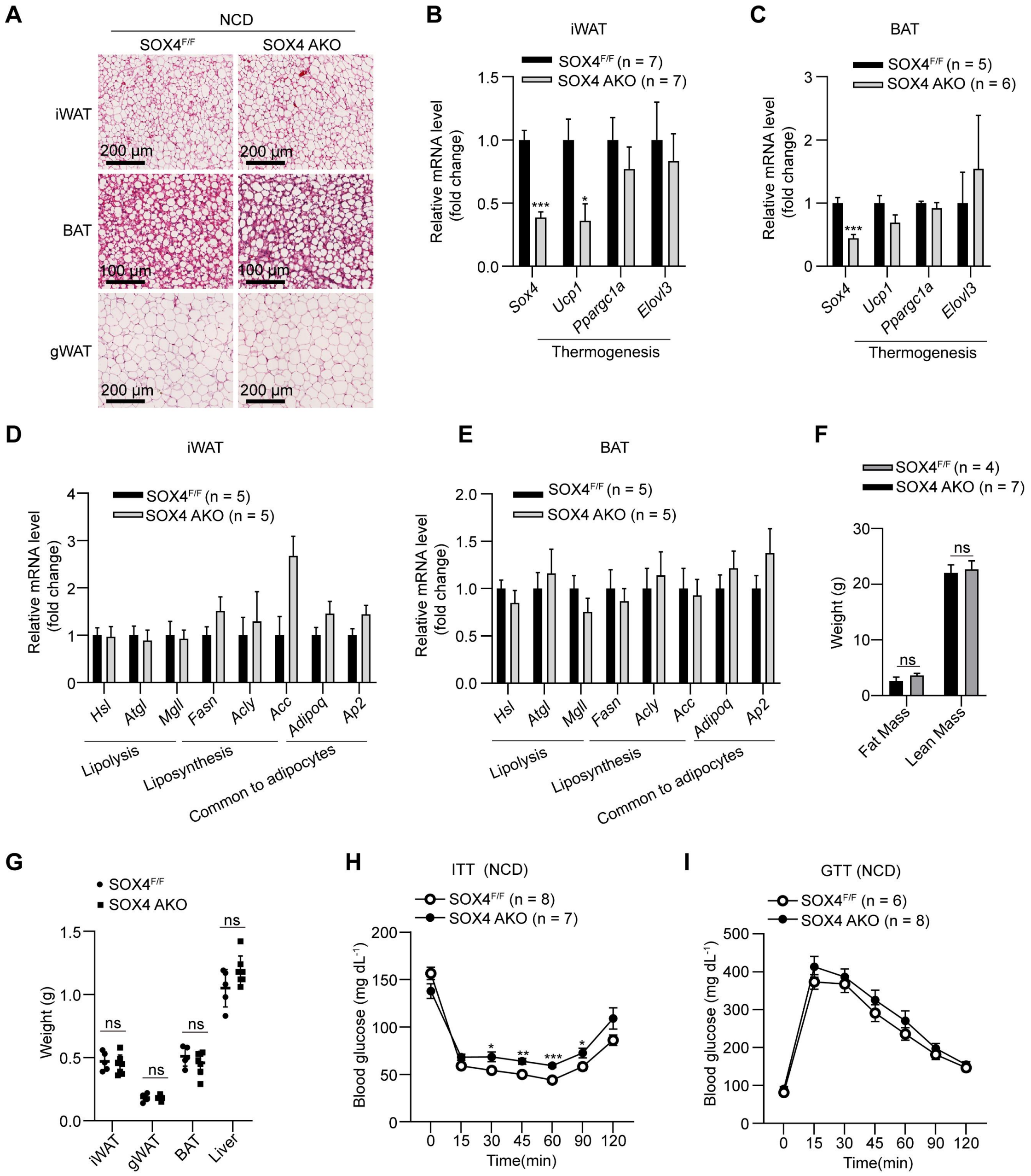

Figure S6. The role of SOX4 in lipid metabolism and homeostasis under NCD and room temperature.

10-week control and SOX4 AKO male mice were housed at room temperature and fed with NCD. **(A)** Representative images of H&E staining of iWAT, gWAT and BAT were shown. Scale bars, as indicated. **(B-C)** mRNA levels of thermogenic genes in the iWAT (B) and BAT (C) were shown. **(D-E)** mRNA levels of lipolytic, lipogenic and common adipogenic genes in the iWAT (D) and BAT (E) were shown. **(F-G)** The average fat and lean mass (F), and weights of iWATs, gWATs, BATs and Livers (G) are shown. **(H-I)** ITT (H) performed on 10-week-old male mice and GTT (I) performed on 11-week-old male mice. The blood glucose levels were measured within 2 h after insulin (H) and glucose (I) injections.

Figure S7

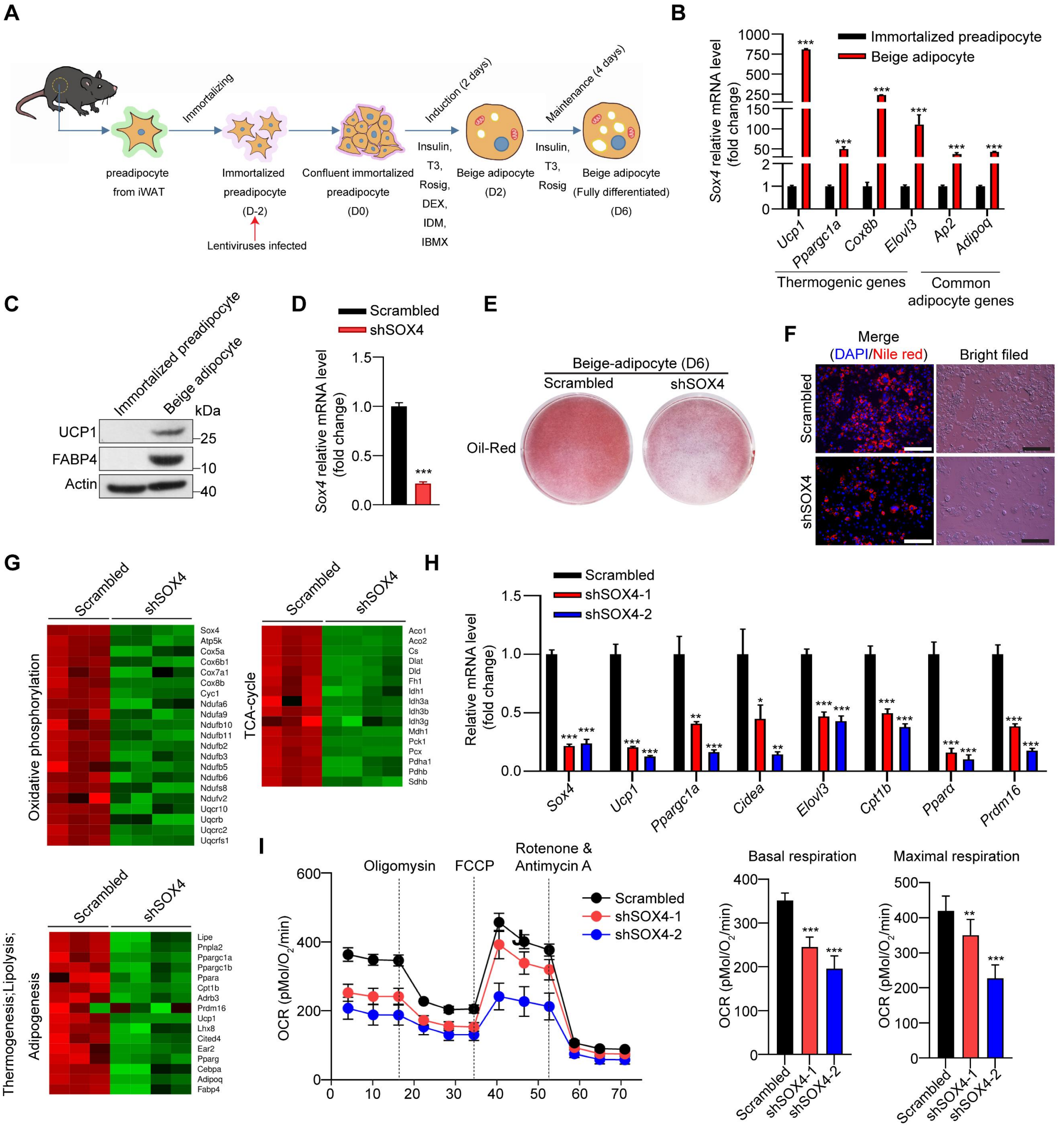

Figure S7. Sox4 KD inhibits beige adipocyte differentiation. (A) Schematic illustration of differentiation of beige adipocytes with lentiviral infection in vitro. **(B)** qPCR analysis of mRNA levels of indicated genes in immortalized preadipocytes and beige adipocytes at day 6 post of differentiation. **(C)** Western blotting showing the protein levels of UCP1 in immortalized preadipocytes and beige adipocytes (day 6). **(D)** mRNA levels of Sox4 in scrambled and shSOX4 beige adipocytes. **(E-F)** Oil-red-O staining (E) and Nile-red (F, left) staining were performed at day 6 post of differentiation. Scale bar, 100 μm. **(G)** Heatmap of the RNA-Seq showed down-regulated genes in beige adipocytes with Sox4 knockdown. a cutoff of fold change ≥ 2, p value < 0.05. **(H)** qPCR analysis of mRNA levels of indicated genes in the scrambled and shSOX4 beige adipocytes at day 6 of differentiation. lentiviral infection as (A). **(I)** Immortalized preadipocytes were infected with scrambled and shSOX4 lentiviruses and analyzed for OCR at day 6 of differentiation. Oligomycin, FCCP, and

rotenone/antimycin A were added at the time points indicated by dashed lines. Right panels showed averaged basal and maximal respiration rates, respectively.

Figure S8

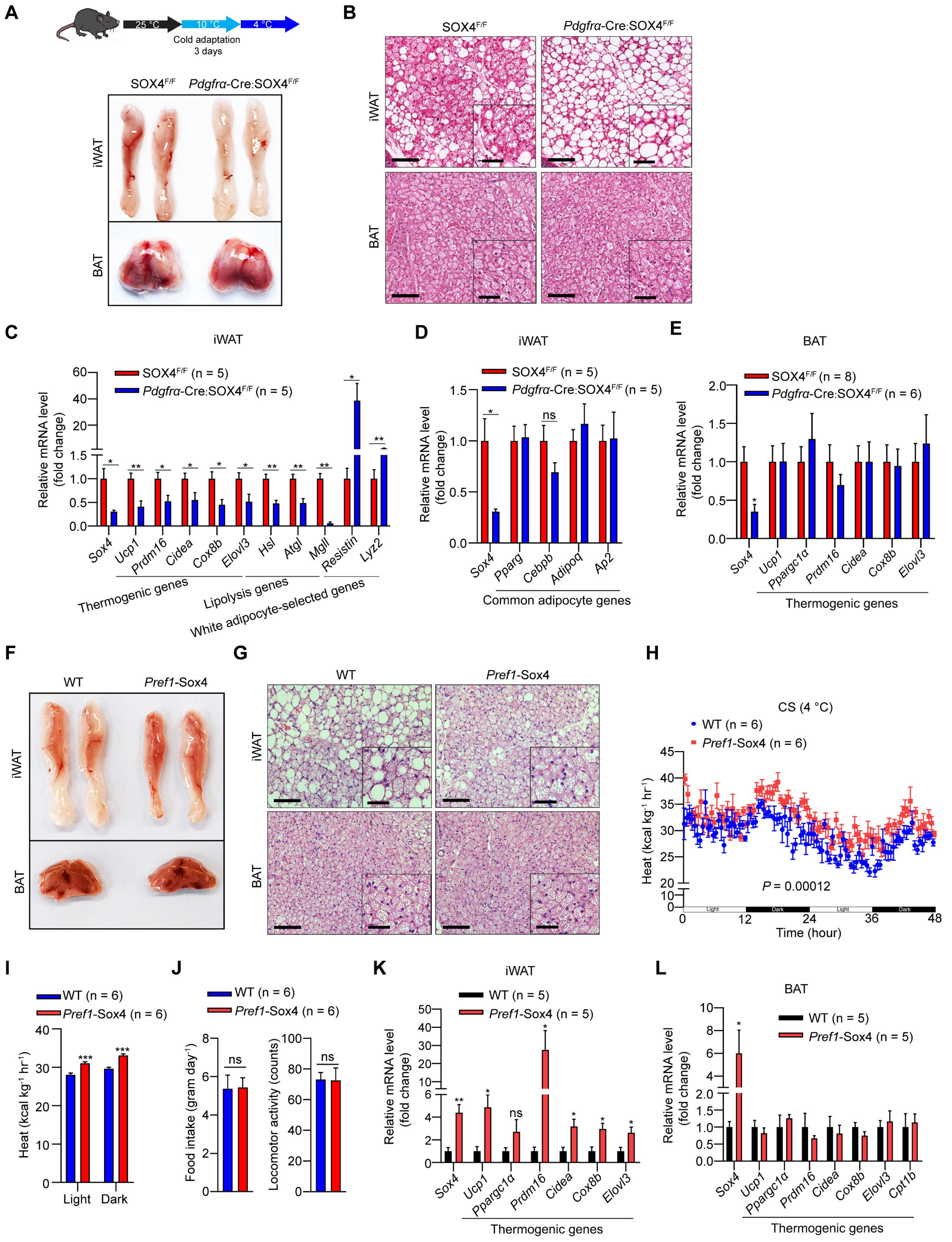

Figure S8. SOX4 promotes the biogenesis of beige adipocyte and the expression of thermogenic genes. (A-E) 10-week-old Pdgfrα-Cre: SOX4$^{F/F}$ mice and control littermates were exposed to 25 °C for 3 days, then 10 °C for 3 days and 4 °C for 3 days. **(A-B)** Representative image (A) and H&E staining (B) of iWAT and BAT were shown. Scale bar, 100 μm. Insets show higher magnification, scale bar, 50 μm. **(C-E)** qPCR analysis of

indicated genes mRNA expression in the iWAT (C-D) and BAT (E). **(F-L)** 8-week-old Pref1-Sox4 mice and control littermates were exposed to 25 °C for 3 days, then 10 °C for 3 days and 4 °C for 3 days. **(F-G)** Representative image (F) and H&E staining (G) of iWAT and BAT were shown. Scale bar, 100 μm. Insets show higher magnification, scale bar, 50 μm. **(H-J)** Heat production (H-I), food intake and locomotor activity (J) were shown. **(K-L)** The relative mRNA levels of indicated genes in the iWAT (K) and BAT (L).

Figure S9

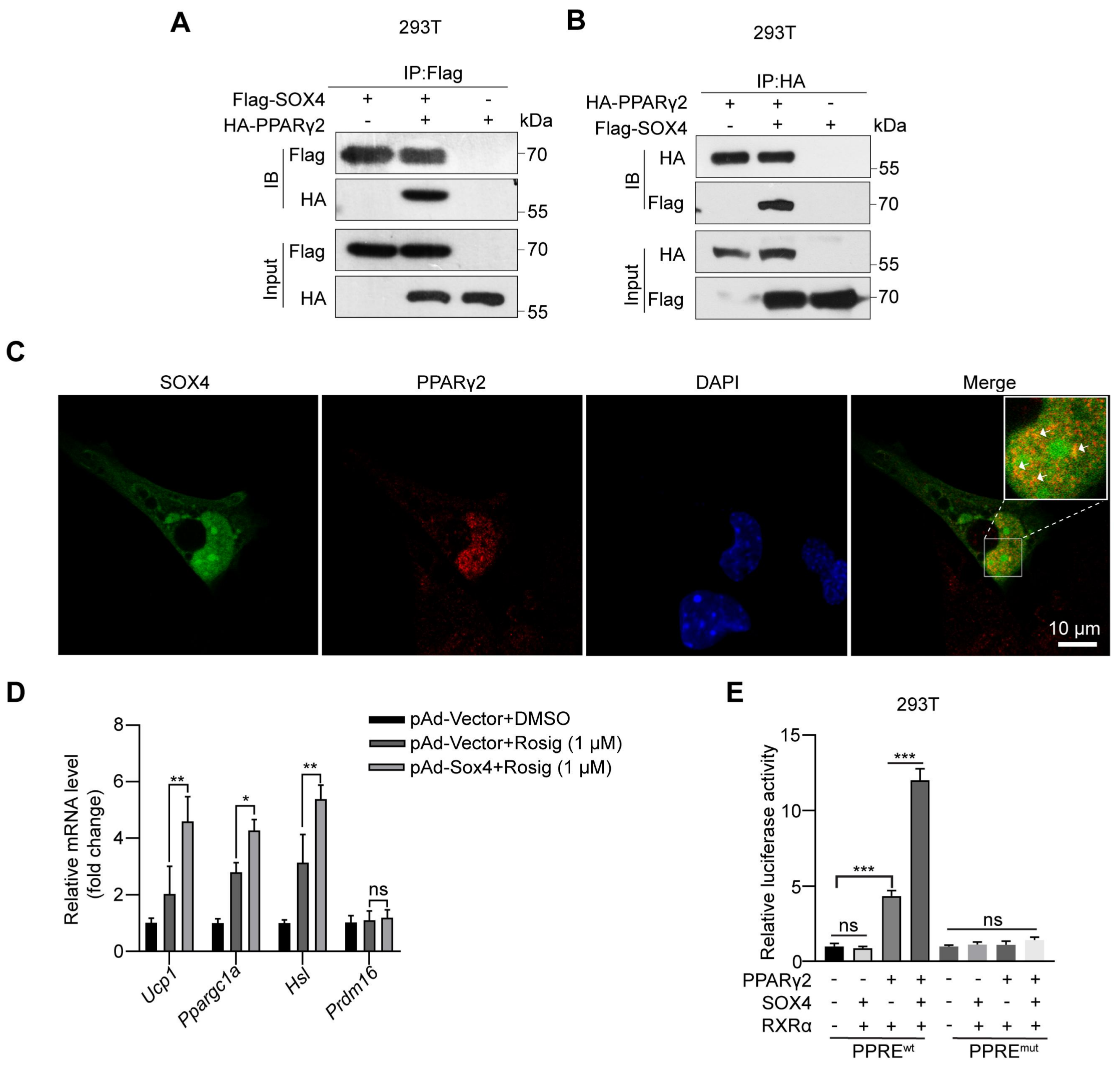

Figure S9. SOX4 activates the transcription activity of Ucp1 by cooperating with PPARγ2. (A-B) HEK293T were transfected with Flag-SOX4 and HA-Pparγ2 as indicated. 48 hr after transfection, cells were lysed and subjected into immunoprecipitation with anti-Flag (A) or anti-HA (B) antibody followed by Western blot. **(C)** Immunofluorescence analysis showed SOX4 colocalized with PPARγ2 in the nucleus of mature beige adipocyte (D6). Scale bar, 10μm. **(D)** Beige adipocytes (day 4) differentiated from immortalized preadipocytes were infected with Vector or Sox4-expression adenovirus. On day 6, cells were treated with or without rosig (1 μM) for 5 hr. The relative mRNA levels of indicated genes were shown. **(E)** The Ucp1 enhancer containing PPRE site or mutated PPRE site was cloned into pGL4.26-basic vector and co-transfected into HEK293T cells together with β-gal and RXRα in the presence or absence of PPARγ2 or SOX4 expression plasmid. Luciferase activity was corrected for corresponding β-gal activity and normalized to control activity.

Figure S10

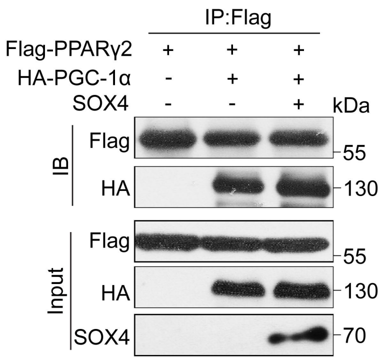

Figure S10. SOX4 did not affect the interaction of PPARγ2 and PGC1α. HEK293T were transfected with Flag-Pparγ2, HA-PGC-1α, and SOX4-GFP as indicated. 48 hr after transfection, cells were lysed and subjected into immunoprecipitation with anti-Flag antibody followed by Western blot.

Table S1. Sequences of primers for qPCR analysis

Name	Forward (5'-3' sequence)	Reverse (5'-3' sequence)
m18s	GTCTGTGATGCCCTTAGATG	AGCTTATGACCCGCACTTAC
mSox4	CGGCTGCATCGTTCTCTCC	CGCTTCACTTTCTTGTCGGC
mUcp1	ACCACCCTGGCAAAAACAGA	CCTCTGTAGGCTGCCCAATG
mPpargc1α	GCACTTCGGTCATCCCTGTC	GGCGACACATCGAACAATGA
mCidea	CAAGGTCGGGTCAAGTCGTC	GGGCGAGCTGGATGTATGAG
mCox5b	GCTGCATCTGTGAAGAGGACAAC	CAGCTTGTAATGGGTTCCACAGT
mElovl3	TGGAAGGACAGAGGCACACA	ACAGCCGGTAGGTCTGGTCA
mCpt1b	GCACACCAGGCAGTAGCTTT	CAGGAGTTGATTCCAGACAGGTA
mPrdm16	CGCGGAAGAACCACGTCTAC	TGCCACCTTCCGCTTTTCTA
mHsl	AAGGACTTGAGCAACTCAGA	TTGACTATGGGTGACGTGTA
mAtgl	CATGATGGTGCCCTATACTC	GTGAGAGGTTGTTTCGTACC
mMgll	GACGGACAGTACCTCTTTTG	AGAAAAGTAGGTTGGCCTCT
mCox8b	AAGCCCATGTCTCTGCCAAG	CTTCATGCTGCGGAGCTCTT
mDio2	GAAGCAGAGTGCCCAGGAGA	CCACGTGCTTGAGCAGAATG
mPparα	GCGTACGGCAATGGCTTTAT	GAACGGCTTCCTCAGGTTCTT
mAdipoq	TGTTCCTCTTAATCCTGCCCA	CCAACCTGCACAAGTTCCCTT
mPparγ	TGGCATCTCTGTGTCAACCATG	GCATGGTGCCTTCGCTGA
mCebpb	ACGACTTCCTCTCCGACCTCT	CGAGGCTCACGTAACCGTAGT
mAp2	CTGGTGCAGGTGCAGAAGTG	TCCATCCAGGCCTCTTCCTT
mResistin	AAGAACCTTTCATTTCCCCTCCT	GTCCAGCAATTTAAGCCAATGTT
mAgt	AAGACCCTGCATGATCAGCTC	CTTCCTGCCTCATTCAGCATC
mLyz2	GAATGGAATGGCTGGCTACT	CGTGCTGAGCTAAACACACC
mF4/80	TTTCCTCGCCTGCTTCTTC	CCCCGTCTCTGTATTCAACC
mMcp1	ATGCAGGTCCCTGTCATGCTT	GGCATCACAGTCCGAGTCACAC
mIl6	CTGATGCTGGTGACAACCAC	TTTTCTGCAAGTGCATCATCGT
mTnfa	ACACTCAGATCATCTTCTCAAAATTCG	GTGTGGGTGAGGAGCACGTAGT
mCd11c	AAAATCTCCAACCCATGCTG	CACCACCAGGGTCTTCAAGT
mCd206	CAAGGAAGGTTGGCATTTGT	CCTTTCAGTCCTTTGCAAGC
mCox7a1	GCTCTGGTCCGGTCTTTTAGC	GTACTGGGAGGTCATTGTCGG
mAtp8	TCACAGTTCAAGTTCCTGCAAC	GGCTGAGAAACCGCAGAAGAA
mNdufv1	TTTCTCGGCGGGTTGGTTC	GGTTGGTAAAGATCCGGTCTTC
mFasn	GGAGGTGGTGATAGCCGGTAT	TGGGTAATCCATAGAGCCCAG
mAcly	CAGCAGGACAGCATCTTTTTC	TGGACTTGGGACTGAATCTTG
mAcc	AGGAAGATGGTGTCCGCTCTG	GGGGAGATGTGCTGGGTCAT

mCidec	ATGGACTACGCCATGAAGTCT	CGGTGCTAACACGACAGGG
mCpt2	CAGCACAGCATCGTACCCA	TCCCAATGCCGTTCTCAAAAT
mAcox1	TAACTTCCTCACTCGAAGCCA	AGTTCCATGACCCATCTCTGTC
mScad	TGGCGACGGTTACACACTG	GTAGGCCAGGTAATCCAAGCC

Table S2. Targeted sequence of shRNAs

Name	Sequence
mSox4 shRNA-1	GCGAGATGATCTCGGGAGATT
mSox4 shRNA-2	TGAAGCGCGTCTACCTGTTTG
mPparγ shRNA-1	GCTCCACACTATGAAGACATT
mPparγ shRNA-2	GCCCTGGCAAAGCATTTGTAT

Table S3. Sequences of primers for ChIP-qPCR analysis

Name	Forward (5'-3' sequence)	Reverse (5'-3' sequence)
mUcp1	AAGCTTGCTGTCACTCCTCTAC	TCTA GAGTCTGAGGAAAGGG

m: mouse
